# Frame Non-Orthogonality Calibration and Its Application to Transfer Alignment for Hybrid Inertial Navigation Systems

**DOI:** 10.3390/s26217022

**Published:** 2026-11-06

**Authors:** Xiaohan Wang, Chunlei Song, Yuning Jiao, Guoxiang Rao, Jianhua Xu

**Affiliations:** 1 School of Automation, Beijing Institute of Technology, Beijing 100081, China; 18101355235@163.com (X.W.); songchunlei@bit.edu.cn (C.S.); 3220251190@bit.edu.cn (Y.J.); 2 National Key Laboratory of Inertial Measurement, Beijing Institute of Aerospace Control Devices, Beijing 100081, China; 18636168977@163.com

**Keywords:** hybrid inertial navigation system, frame non-orthogonality calibration, recursive least squares (RLS), transfer alignment

## Abstract

Hybrid inertial navigation systems (HINSs) integrate physical, stabilized platforms, strapdown attitude algorithms, and rotation modulation technology, offering unique performance advantages. However, the non-ideal orthogonality of the gimbal frames in the rotation mechanism introduces frame non-orthogonality errors, which limit the accuracy of high-precision attitude extraction and transfer alignment. To address the limitations of existing calibration methods—such as incomplete modeling, poor noise immunity, or dependence on the initial mounting attitude—this paper establishes a complete frame non-orthogonality error model and proposes two improved calibration methods: a three-position self-calibration method and a recursive least squares (RLSs) calibration method. Monte Carlo simulations show that the three-position method is simple in principle but sensitive to sensor measurement noise (accelerometer noise and gyroscope angular random walk), whereas the RLS method recursively fuses observation information and reduces the average standard deviation of the estimated non-orthogonality angles to 0.044″, with superior stability and repeatability. On this basis, the effect of non-orthogonality angle compensation on transfer alignment accuracy is verified, showing that the improvement of horizontal misalignment angles exceeds 96%. Finally, the proposed methods are validated through physical experiments on a HINS. After compensation with the RLS method, the peak residual roll attitude error decreases from 18.53″ to 10.6″, effectively improving the overall navigation performance of the system.

## 1. Introduction

A hybrid inertial navigation system (HINS) is a gimbaled inertial navigation architecture that integrates a physical, stabilized platform, strapdown attitude computation, and rotation modulation in a single system, thereby combining the respective advantages of platform, strapdown, and rotary inertial navigation systems [1]. The introduction of rotation, however, also introduces drawbacks. Because the gimbal frames of the indexing mechanism cannot be installed perfectly orthogonally, frame non-orthogonality angles are inevitably introduced [2]. High-precision attitude information is essential for long-endurance autonomous navigation, and accurate attitude extraction cannot be achieved without accounting for these errors [3]. Nevertheless, most calibration schemes assume that the rotation axes are mutually orthogonal, neglecting the effects of frame non-orthogonality angles and ultimately degrading navigation accuracy.

In addition, frame non-orthogonality errors significantly affect transfer alignment accuracy—the process by which a slave inertial navigation system on a moving platform receives initial attitude and velocity reference information from a higher-accuracy master INS. Traditional marine vehicles often adopt photoelectric aiming for initial alignment, transferring the attitude angles of the master INS to the slave INS using optical devices such as collimator tubes, self-calibrating theodolites, and prisms. Although this approach achieves high alignment accuracy, it requires complex hardware, occupies substantial space, and is costly. The current trend toward transfer alignment relies instead on attitude-matching and velocity-matching methods [4]. For a strapdown slave INS, the attitude angles produced by the navigation solution can be directly compared with those of the master INS. For a HINS slave INS, however, the attitude angles output by the navigation solution are relative to the platform body and must first be transformed into the carrier coordinate system before attitude matching. Non-orthogonality errors of the gimbal frames introduce substantial errors during this transformation—on the order of arc-minutes—and thus significantly affect transfer alignment accuracy. Suppressing these errors is therefore of practical importance for improving the transfer alignment and navigation accuracy of HINS.

Existing methods for calibrating frame non-orthogonality angles in rotary and hybrid inertial navigation systems can be divided into two categories: fitting-filtering methods and analytical methods. Fitting-filtering methods calibrate non-orthogonality angles by establishing the mapping relationship between non-orthogonality angles and navigation output errors using filtering or parameter identification algorithms. For single-axis rotary INS, Wang Kunming et al. proposed a Kalman filtering non-orthogonality calibration method for field conditions with swaying interference [5]. Ye et al. presented an RLS calibration method using both accelerometer integral outputs and attitude errors as observations [6]. For dual-axis rotary INS, Wang et al. proposed an extended Kalman filtering-based calibration method, which systematically calibrates 25-dimensional errors but only considers one non-orthogonality angle [7]. Lin et al. established an error model containing four non-orthogonality angles and realized systematic error calibration using a 31-dimensional Kalman filter with velocity and attitude errors as observations, ensuring full system observability, but lacked physical experimental verification [8]. Bai et al. constructed a dual-filter model to avoid complex separation processes but did not consider non-orthogonality angles between inner and outer axes [9]. Wei et al. proposed a self-calibration scheme capable of calibrating all non-orthogonality angles simultaneously by constructing a Kalman filter with attitude variation as the observation, based on the property that attitude angles remain constant when the carrier is stationary [10]. For three-axis rotary INS, Gao et al. put forward a two-step self-calibration scheme based on RLS, which efficiently calibrates three non-orthogonality angles and part of gyro installation errors using attitude and velocity errors as observations but relies on the initial placement position [11]. Liu et al. established a comprehensive error model including frame non-orthogonality errors, encoder installation errors and system null errors and calibrated them using the RLS method [12]. Recently, Sun et al. proposed an invariant-error-based fast self-calibration method for dual-axis RINS that completes calibration within 12 min [13], and Liu et al. developed a dual-axis non-orthogonality calibration scheme without requiring special alignment procedures [14].

Analytical methods solve frame non-orthogonality angles by establishing direct algebraic relationships between frame non-orthogonality angles and sensor outputs or attitude matrices using observation results. For dual-axis rotary INS, Deng et al. proposed a simple and fast two-position rotary self-calibration method to calibrate six non-orthogonality angles, but some frame non-orthogonality angles had poor observability and could not be fully estimated [15]. Lin et al. simplified frame non-orthogonality angles, but the calibration accuracy decreased compared with filtering methods [16]. For three-axis rotary INS, Liu et al. proposed a self-calibration method based on gyro and angle encoder outputs, considering the influence of temperature on non-orthogonality angles. A temperature compensation model for non-orthogonality angles was established using a second-order polynomial equation, but the fitting accuracy of this model was insufficient for two of the non-orthogonality angles [17].

In summary, existing non-orthogonality calibration methods still have the following limitations: recent works such as the collaborative calibration of redundant dual-axis RINS under geometric constraints [18] have advanced the field, but most studies focus on dual-axis rotary INS, with relatively little work on three-axis and hybrid systems; some methods use incomplete or redundant error models and neglect the impact of non-orthogonality angles on transfer alignment; analytical methods underuse the available observation data, leading to large dispersion and poor repeatability, while fitting-filtering methods suffer from high model complexity as the number of states increases and rely on a specific initial mounting attitude.

Recent works have advanced rotary INS calibration: Sun et al. [13] proposed a fast self-calibration method based on invariant errors, Liu et al. [14] developed a dual-axis RINS self-calibration approach, and Liang et al. [18] introduced collaborative calibration for redundant dual-axis RINS using geometric constraints. However, these methods primarily address dual-axis RINS; the frame non-orthogonality model and three-position/RLS calibration for HINS with a three-axis physical platform remain underexplored.

To address the high-precision calibration of frame non-orthogonality angles, this paper establishes a complete frame non-orthogonality error model and analyzes its influence on navigation accuracy. Drawing on both analytical and fitting-filtering ideas, two improved calibration methods are proposed: the three-position self-calibration method and the RLS calibration method. Using a HINS as the slave INS, we study how compensation for frame non-orthogonality errors affects transfer alignment. The effectiveness of the proposed methods is verified through both simulations and physical experiments. The remainder of this paper is organized as follows: Section 2 derives the error model of the HINS and the frame non-orthogonality model, analyzes their influence on navigation accuracy, and details the principles of the two calibration methods. Section 3 evaluates their performance through Monte Carlo simulations, examines the effect of non-orthogonality compensation on transfer alignment, and presents the physical experimental results. Section 4 draws the conclusions.

## 2. Materials and Methods

### 2.1. Error Model of HINS and Coordinate System Definition

A hybrid inertial navigation system employs a three-axis physical platform that isolates the carrier’s angular motion and Earth rotation and is fixed to the base via three gimbal axes [19]. The inertial sensors are mounted on the inner shaft, which is attached to the middle gimbal axis; the middle gimbal axis is connected to the outer gimbal axis, and the outer gimbal axis is fixed to the carrier base. For navigation computation and error analysis, the commonly used coordinate frames in the HINS are defined in Table 1. In this paper, the body frame (b) is the vehicle/carrier frame fixed to the marine vehicle; the IMU frame (s) is aligned with the sensor triad axes; and the gimbal frames h1, h2, h3 denote the base, inner, and outer gimbal frames, respectively.

The non-orthogonal errors of the hybrid inertial navigation system mainly originate from two sources: the installation non-orthogonality errors of the inertial measurement unit (IMU) and the frame non-orthogonality errors of the indexing mechanism, which are coupled with each other. Therefore, before compensating for the frame non-orthogonality errors, it is necessary to model, calibrate, and compensate for the inertial sensor errors first. The error equations of the gyroscope and accelerometer under linear approximation are given by the following formulas.

(1) $$\delta \omega_{g}={\tilde{\omega }}_{\mathrm{is}}^{s}-\omega_{\mathrm{is}}^{s}=\left(K_{g}+C_{s}^{g}\right)\omega_{\mathrm{is}}^{s}+\varepsilon_{g}+K_{g2}\left|\omega_{\mathrm{is}}^{s}\right|+{\bar{\omega }}_{g}^{s}$$

(2) $$\delta f_{s}={\tilde{f}}_{s}-f_{s}=\left(K_{a}+C_{s}^{a}\right)f_{s}+\nabla_{a}+K_{a2}\left|f_{s}\right|+{\bar{\omega }}_{a}^{s}$$

where $\omega_{\mathrm{is}}^{s}$ is the theoretical value of the gyroscope’s input angular velocity, and ${\tilde{\omega }}_{\mathrm{is}}^{s}$ is the measured value of the output angular velocity. *K_g_*, $C_{s}^{g}$, *ε_g_*, and *K_g_*_2_ represent the scale factor error, installation error, constant drift, and positive/negative scale factor bias of the gyroscope, respectively. ${\bar{\omega }}_{g}^{s}$ denotes the gyroscope noise. *f_s_* is the theoretical value of the accelerometer’s input specific force, and ${\tilde{f}}_{s}$ is the measured value of the accelerometer’s output specific force. *K_a_*, $C_{s}^{a}$, ∇*_a_*, and *K_a_*_2_ represent the scale factor error, installation error, constant bias, and positive/negative scale factor bias of the accelerometer, respectively. ${\bar{\omega }}_{a}^{s}$ denotes the accelerometer noise.

### 2.2. Frame Non-Orthogonal Angles of HINS

**Figure 1:**
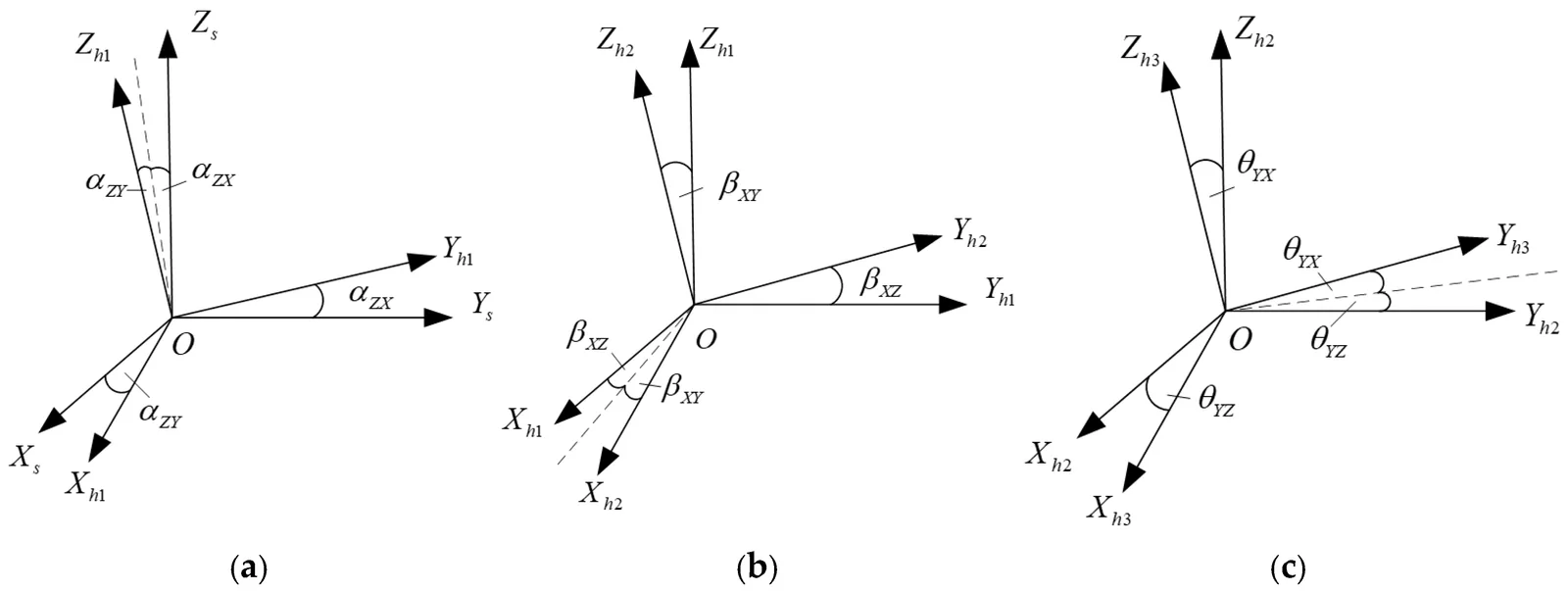
Definition diagram of frame non-orthogonality angles. (**a**) Platform-axis (s–h1) non-orthogonality angles. (**b**) Inner-gimbal-axis (h1–h2) non-orthogonality angles. (**c**) Outer-gimbal-axis (h2–h3) non-orthogonality angles.

The three-axis physical platform of the hybrid inertial navigation system is rigidly mounted to the base via three gimbal axes: the platform axis, the inner gimbal axis, and the outer gimbal axis, which form the h1, h2, and h3 coordinate frames, respectively. Among these, the *z_h_*_1_, *x_h_*_2_, and *y_h_*_3_ axes correspond to the inner motor axis, middle motor axis, and outer motor axis of the HINS. The definition of frame non-orthogonality angles is shown in Figure 1. Six parameters are interpreted as signed small rotation components under the convention used in Equation (6): *α_ZX_* is the small rotation component about the local X-axis in the platform-axis (s–h1) correction; *α_ZY_* is the small rotation component about the local Y-axis in the platform-axis (s–h1) correction; *β_XY_* is the small rotation component about the local Y-axis in the inner-gimbal-axis (h1–h2) correction; *β_XZ_* is the small rotation component about the local Z-axis in the inner-gimbal-axis (h1–h2) correction; *θ_YX_* is the small rotation component about the local X-axis in the outer-gimbal-axis (h2–h3) correction; *θ_YZ_* is the small rotation component about the local Z-axis in the outer-gimbal-axis (h2–h3) correction. Each pair describes the two transverse components of the correction associated with the corresponding nominal rotation axis.

When frame non-orthogonality errors are not considered, the ideal rotation matrix ${\bar{C}}_{s}^{b}$ can be expressed as

(3) $${\bar{C}}_{s}^{b}=R_{y}\left(\varphi_{y}\right)R_{x}\left(\varphi_{x}\right)R_{z}\left(\varphi_{z}\right)$$

(4) $$\begin{array}{c} R_{y}\left(\varphi_{y}\right)=\left[\begin{array}{ccc} \cos\varphi_{y} & 0 & \sin\varphi_{y}\\ 0 & 1 & 0\\ -\sin\varphi_{y} & 0 & \cos\varphi_{y} \end{array}\right]\;\;R_{x}\left(\varphi_{x}\right)=\left[\begin{array}{ccc} 1 & 0 & 0\\ 0 & \cos\varphi_{x} & -\sin\varphi_{x}\\ 0 & \sin\varphi_{x} & \cos\varphi_{x} \end{array}\right]\\ R_{z}\left(\varphi_{z}\right)=\left[\begin{array}{ccc} \cos\varphi_{z} & -\sin\varphi_{z} & 0\\ \sin\varphi_{z} & \cos\varphi_{z} & 0\\ 0 & 0 & 1 \end{array}\right] \end{array}$$

where *φ_y_*,*φ_x_*,*φ_z_* are the rotation angles of the outer gimbal axis, inner gimbal axis, and platform axis, respectively. Due to the inability to achieve perfect orthogonal installation between the frames, the influence of frame non-orthogonality angles must be considered. When considering the six frame non-orthogonality angles defined in the previous section, the rotation matrix $C_{s}^{b}$ can be expressed as:

(5) $$C_{s}^{b}=R_{y}\left(\varphi_{y}\right){R^{\prime }}_{y}R_{x}\left(\varphi_{x}\right){R^{\prime }}_{x}R_{z}\left(\varphi_{z}\right){R^{\prime }}_{z}$$

Since all non-orthogonality angles are small angles, the frame non-orthogonality projection matrix can be obtained based on the small-angle approximation as follows.

(6) $$\begin{array}{c} {R^{\prime }}_{y}=\left[\begin{array}{ccc} 1 & -\theta_{\mathrm{YZ}} & 0\\ \theta_{\mathrm{YZ}} & 1 & -\theta_{\mathrm{YX}}\\ 0 & \theta_{\mathrm{YX}} & 1 \end{array}\right]\;\;{R^{\prime }}_{x}=\left[\begin{array}{ccc} 1 & -\beta_{\mathrm{XZ}} & \beta_{\mathrm{XY}}\\ \beta_{\mathrm{XZ}} & 1 & 0\\ -\beta_{\mathrm{XY}} & 0 & 1 \end{array}\right]\\ {R^{\prime }}_{z}=\left[\begin{array}{ccc} 1 & 0 & \alpha_{\mathrm{ZY}}\\ 0 & 1 & -\alpha_{\mathrm{ZX}}\\ -\alpha_{\mathrm{ZY}} & \alpha_{\mathrm{ZX}} & 1 \end{array}\right] \end{array}$$

Here, ${R^{\prime }}_{z}$ is the small-angle projection matrix between the IMU frame (s) and the platform frame (h1), composed of the two platform-axis non-orthogonality angles *α_ZX_* and *α_ZY_*. ${R^{\prime }}_{x}$ is the projection matrix between the platform frame (h1) and the inner gimbal frame (h2), composed of *β_XZ_* and *β_XY_*. ${R^{\prime }}_{y}$ is the projection matrix between the inner gimbal frame (h2) and the outer gimbal frame (h3), composed of *θ_YZ_* and *θ_YX_*. The multiplication order in Equation (5) follows the frame cascade from s to b (platform → inner gimbal → outer gimbal), so that each ideal rotation *R_i_* is preceded by the corresponding non-orthogonality projection ${R^{\prime }}_{i}$.

### 2.3. Influence of Frame Non-Orthogonality Angles on Navigation Accuracy

At the start of calibration, each gimbal is aligned to its mechanical reference position (encoder home position and mechanical limit stops) so that the encoder rotation angles of the platform axis, inner gimbal axis, and outer gimbal axis are all set to 0°. This is a mechanical initialization step, not an attitude-leveling requirement. Deviations from this zero-angle encoder position are negligible because the calibration algorithm operates on angular increments relative to this reference, and the small-angle linearization remains valid even if the residual offset is on the order of a few arcminutes. Any residual initial encoder offset is absorbed into the constant terms of the observation model and does not bias the estimated non-orthogonality angles.

Assume that the platform axis, inner gimbal axis, and outer gimbal axis inside the hybrid inertial navigation system rotate with angular velocities *ω_z_*,*ω_x_*,*ω_y_* respectively. Based on the chain transfer rule, the angular velocity under ideal conditions ${\bar{\omega }}_{\mathrm{bs}}^{s}$ and the angular velocity including non-orthogonality errors $\omega_{\mathrm{bs}}^{s}$ can be expressed as

(7) $${\bar{\omega }}_{\mathrm{bs}}^{s}=\omega_{z}+{R_{z}}^{-1}\omega_{x}+{R_{z}}^{-1}{R_{x}}^{-1}\omega_{y}$$

(8) $$\omega_{\mathrm{bs}}^{s}={{R^{\prime }}_{z}}^{-1}\omega_{z}+{{R^{\prime }}_{z}}^{-1}{R_{z}}^{-1}{{R^{\prime }}_{x}}^{-1}\omega_{x}+{{R^{\prime }}_{z}}^{-1}{R_{z}}^{-1}{{R^{\prime }}_{x}}^{-1}{R_{x}}^{-1}{{R^{\prime }}_{y}}^{-1}\omega_{y}$$

Assuming the carrier is stationary, both the carrier attitude angular velocity $\omega_{\mathrm{nb}}^{n}$ and the transport angular velocity $\omega_{\mathrm{en}}^{n}$ caused by the carrier’s motion on the Earth’s surface are zero. When comprehensively considering Earth’s rotation, inertial sensor errors, and frame non-orthogonality errors, the gyroscope angular velocity measurement ${\tilde{\omega }}_{\mathrm{is}}^{s}$ is given by Equation (9). where *ε* denotes the gyroscope constant drift (bias) vector.

(9) $$\begin{array}{rcl} {\tilde{\omega }}_{\mathrm{is}}^{s} & = & \left(I+K_{g}+C_{s}^{g}\right)C_{n}^{s}\omega_{\mathrm{is}}^{n}+\varepsilon \\ & = & \left(I+K_{g}+C_{s}^{g}\right)\left(C_{n}^{s}\omega_{\mathrm{ie}}^{n}+\omega_{\mathrm{bs}}^{s}\right)+\varepsilon \end{array}$$

When only inertial sensor errors are considered, and substituting ${\bar{C}}_{n}^{s}={\bar{C}}_{b}^{s}C_{n}^{b}$, the gyroscope angular velocity measurement ${\bar{\omega }}_{\mathrm{is}}^{s}$ is obtained as shown in Equation (10).

(10) $$\begin{array}{rcl} {\bar{\omega }}_{\mathrm{is}}^{s} & = & \left(I+K_{g}+C_{s}^{g}\right)\left({\bar{C}}_{n}^{s}\omega_{\mathrm{ie}}^{n}+{\bar{\omega }}_{\mathrm{bs}}^{s}\right)+\varepsilon \\ & = & \left(I+K_{g}+C_{s}^{g}\right)\left({R_{z}}^{-1}{R_{x}}^{-1}{R_{y}}^{-1}C_{n}^{b}\omega_{\mathrm{ie}}^{n}+\omega_{z}+{R_{z}}^{-1}\omega_{x}+{R_{z}}^{-1}{R_{x}}^{-1}\omega_{y}\right)+\varepsilon \end{array}$$

Therefore, if only frame non-orthogonality errors are considered and projected into the carrier coordinate system, the gyroscope error *δω^b^* can be expressed as

(11) $$\begin{array}{rcl} \delta \omega^{b} & = & C_{s}^{b}\left(\omega_{\mathrm{is}}^{s}-{\bar{\omega }}_{\mathrm{is}}^{s}\right)\\ & = & C_{s}^{b}\left({{R^{\prime }}_{z}}^{-1}{R_{z}}^{-1}{{R^{\prime }}_{x}}^{-1}{R_{x}}^{-1}{{R^{\prime }}_{y}}^{-1}{R_{y}}^{-1}C_{n}^{b}\omega_{\mathrm{ie}}^{n}+{{R^{\prime }}_{z}}^{-1}\omega_{z}+{{R^{\prime }}_{z}}^{-1}{R_{z}}^{-1}{{R^{\prime }}_{x}}^{-1}\omega_{x}\right.\\ & + & \left.{{R^{\prime }}_{z}}^{-1}{R_{z}}^{-1}{R_{x}}^{-1}{R_{x}}^{-1}{{R^{\prime }}_{y}}^{-1}\omega_{y}-{R_{z}}^{-1}{R_{x}}^{-1}{R_{y}}^{-1}C_{n}^{b}\omega_{\mathrm{ie}}^{n}-\omega_{z}-{R_{z}}^{-1}\omega_{x}-{R_{z}}^{-1}{R_{x}}^{-1}\omega_{y}\right) \end{array}$$

This error expansion includes both static projection errors associated with the Earth’s rotation and dynamic coupling errors associated with frame rotation. To isolate the influence of frame non-orthogonality angles on navigation accuracy, assume that other inertial sensor errors have already been compensated and consider only the error caused by frame rotation in the presence of non-orthogonality angles. This error can be decomposed into the sum of components contributed independently by the non-orthogonality angles of each axis:

(12) $$\begin{array}{rcl} \delta \omega^{b} & = & \delta_{z}+\delta_{x}+\delta_{y}\\ & = & R_{y}{R^{\prime }}_{y}R_{x}{R^{\prime }}_{x}R_{z}\left(I-{R^{\prime }}_{z}\right)\omega_{z}+R_{y}{R^{\prime }}_{y}R_{x}\left(I-{R^{\prime }}_{x}R_{z}{R^{\prime }}_{z}{R_{z}}^{-1}\right)\omega_{x}\\ & + & R_{y}\left(I-{R^{\prime }}_{y}R_{x}{R^{\prime }}_{x}R_{z}{R^{\prime }}_{z}{R_{z}}^{-1}{R_{x}}^{-1}\right)\omega_{y} \end{array}$$

Assume rotations about only the z-axis, x-axis, and y-axis, with angular velocities given by $\omega_{z}={\left[\begin{array}{ccc} 0 & 0 & \Omega_{z} \end{array}\right]}^{T}$, $\omega_{x}={\left[\begin{array}{ccc} \Omega_{x} & 0 & 0 \end{array}\right]}^{T}$, and $\omega_{y}={\left[\begin{array}{ccc} 0 & \Omega_{y} & 0 \end{array}\right]}^{T}$. Substituting these into Equation (12), the resulting measurement errors when rotating about different axes are shown in Equations (13), (14), and (15), respectively. Here, *ω_z_*, *ω_x_*, and *ω_y_* are angular velocity vectors associated with the respective gimbal rotations; their nonzero components Ω*_z_*, Ω*_x_*, and Ω*_y_* are scalar angular rates.

(13) $$\delta \omega^{b}=\delta_{z}=\left[\begin{array}{c} \left(-\cos\varphi_{z}\alpha_{\mathrm{ZY}}-\sin\varphi_{z}\alpha_{\mathrm{ZX}}\right)\Omega_{z}\\ \left(-\sin\varphi_{z}\alpha_{\mathrm{ZY}}+\cos\varphi_{z}\alpha_{\mathrm{ZX}}\right)\Omega_{z}\\ 0 \end{array}\right]$$

(14) $$\delta \omega^{b}=\delta_{x}=\left[\begin{array}{c} 0\\ \left[-\beta_{\mathrm{XZ}}\cos\varphi_{x}-\left(\beta_{\mathrm{XY}}+\alpha_{\mathrm{ZY}}\right)\sin\varphi_{x}\right]\Omega_{x}\\ \left[-\beta_{\mathrm{XZ}}\sin\varphi_{x}+\left(\beta_{\mathrm{XY}}+\alpha_{\mathrm{ZY}}\right)\cos\varphi_{x}\right]\Omega_{x} \end{array}\right]$$

(15) $$\delta \omega^{b}=\delta_{y}=\left[\begin{array}{c} \left[\left(\theta_{\mathrm{YZ}}+\beta_{\mathrm{XZ}}\right)\cos\varphi_{y}-\left(\theta_{\mathrm{YX}}+\alpha_{\mathrm{ZX}}\right)\sin\varphi_{y}\right]\Omega_{y}\\ 0\\ \left[-\left(\theta_{\mathrm{YZ}}+\beta_{\mathrm{XZ}}\right)\sin\varphi_{y}-\left(\theta_{\mathrm{YX}}+\alpha_{\mathrm{ZX}}\right)\cos\varphi_{y}\right]\Omega_{y} \end{array}\right]$$

The above results show that when the INS rotates about a single axis, the non-orthogonality errors produce sine/cosine modulation errors on the other two non-rotating axes, with amplitudes proportional to the angular rate of the rotating axis. To quantify this effect, assume a rotation rate of 6°/s and a frame non-orthogonality angle of 100″ on each axis. Rotating about the z-axis alone produces a maximum equivalent measurement error of about 14.81″/s; rotating about the x-axis alone raises this to about 23.41″/s; and rotating about the y-axis alone reaches the maximum of about 29.62″/s. Frame non-orthogonality errors therefore have a pronounced effect on navigation accuracy and must be modeled and compensated.

**Figure 2:**
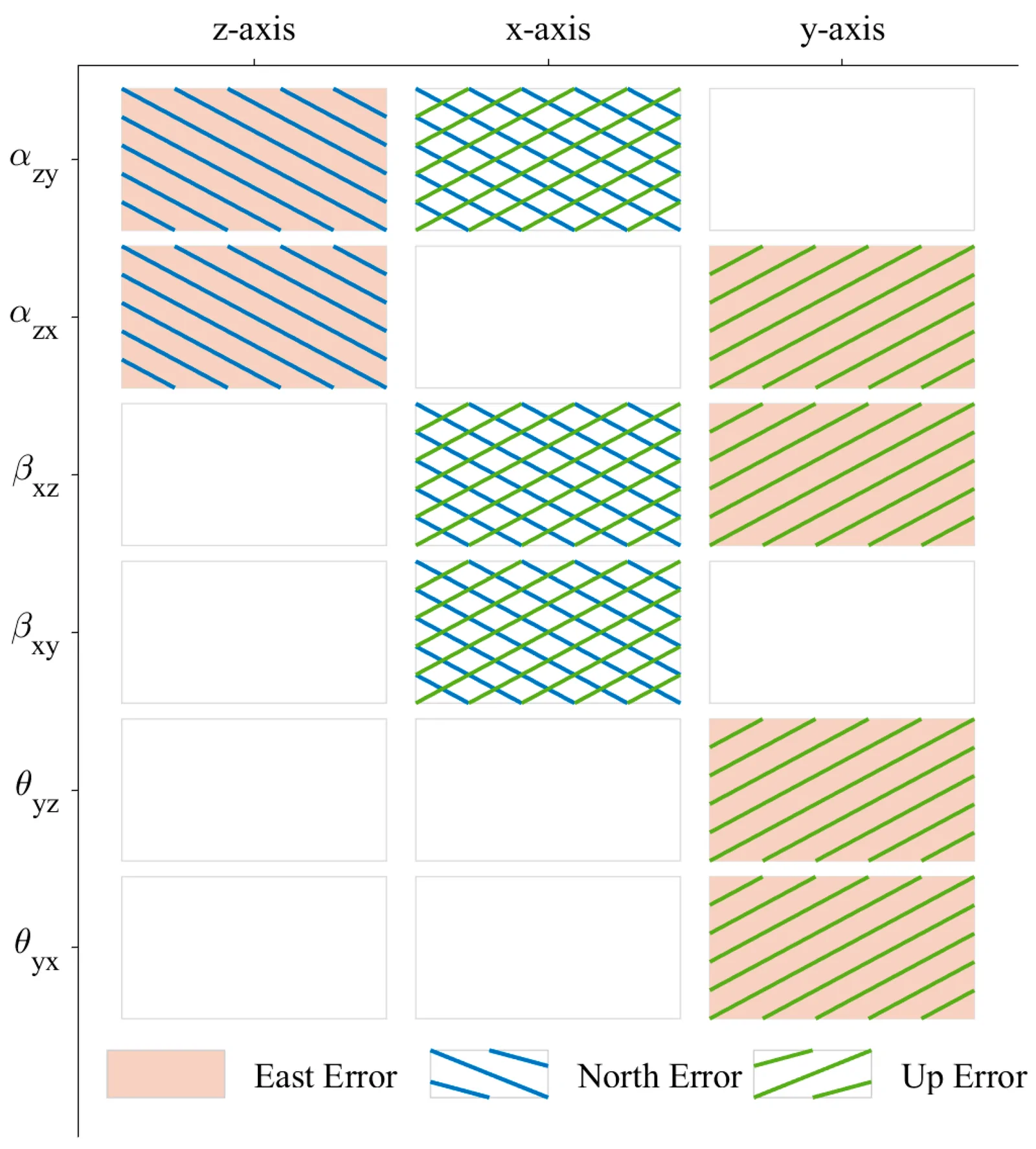
Distribution diagram of excitation errors caused by frame non-orthogonality angles.

Assuming the navigation frame coincides with the body frame, Figure 2 shows the equivalent gyroscope errors in the east, north, and up directions excited by the six non-orthogonality angles under rotations about different axes. No single-axis rotation simultaneously excites all six angles. A high-precision calibration scheme therefore requires a carefully designed multi-axis alternating rotation sequence that excites and identifies all coupling terms.

### 2.4. Three-Position Self-Calibration Method

This paper addresses the problem of frame non-orthogonality error suppression in hybrid inertial navigation systems. Based on the two-position self-calibration method for dual-axis rotational inertial navigation systems in reference [16], a three-position frame non-orthogonality angle self-calibration method suitable for hybrid inertial navigation architectures is derived and proposed. Its core idea is to isolate the motion of individual frame axes and use the constraint that the navigation system attitude should be constant under static carrier conditions to directly solve for the frame non-orthogonality angles from attitude changes. This method features a simple procedure and low computational complexity, enabling rapid estimation of frame non-orthogonality angles. Assuming that at the initial moment, the three-axis rotation angles of the hybrid inertial navigation system are all 0°, the transformation matrix from the navigation coordinate system to the IMU coordinate system is given by

(16) $${\tilde{C}}_{n}^{s}={\tilde{C}}_{b}^{s}C_{n}^{b}={\left(R_{y}\left(0\right){R^{\prime }}_{y}R_{x}\left(0\right){R^{\prime }}_{x}R_{z}\left(0\right){R^{\prime }}_{z}\right)}^{-1}C_{n}^{b}={{R^{\prime }}_{z}}^{-1}{{R^{\prime }}_{x}}^{-1}{{R^{\prime }}_{y}}^{-1}C_{n}^{b}$$

Since the carrier remains stationary throughout the calibration process, the transformation matrix $C_{b}^{n}$ is a constant value. When the three axes are rotated to certain angles, respectively, the transformation matrix $C_{n}^{s}$ is shown in Equation (17) below.

(17) $$C_{n}^{s}=C_{b}^{s}C_{n}^{b}={\left(R_{y}\left(\varphi_{y}\right){R^{\prime }}_{y}R_{x}\left(\varphi_{x}\right){R^{\prime }}_{x}R_{z}\left(\varphi_{z}\right){R^{\prime }}_{z}\right)}^{-1}C_{n}^{b}$$

Based on the analysis in Section 2.3, to excite all non-orthogonality angles, the calibration rotation scheme is designed to make the hybrid inertial navigation system perform independent single-axis rotations about the platform axis, inner gimbal axis, and outer gimbal axis, respectively. When rotating only about the platform axis to a certain angle, i.e., *φ_y_* = *φ_x_* = 0°, we obtain

(18) $${\tilde{C}}_{n}^{s}{\left(C_{n}^{s}\right)}^{-1}={{R^{\prime }}_{z}}^{-1}{{R^{\prime }}_{x}}^{-1}{{R^{\prime }}_{y}}^{-1}C_{n}^{b}C_{b}^{n}R_{y}\left(0\right){R^{\prime }}_{y}R_{x}\left(0\right){R^{\prime }}_{x}R_{z}\left(\varphi_{z}\right){R^{\prime }}_{z}={{R^{\prime }}_{z}}^{-1}R_{z}\left(\varphi_{z}\right){R^{\prime }}_{z}$$

Substituting the above equations and neglecting higher-order small quantities, the following observation matrix is

$$\left[\begin{array}{ccc} \cos\varphi_{z} & -\sin\varphi_{z} & \alpha_{\mathrm{ZY}}\cos\varphi_{z}+\alpha_{\mathrm{ZX}}\sin\varphi_{z}-\alpha_{\mathrm{ZY}}\\ \sin\varphi_{z} & \cos\varphi_{z} & \alpha_{\mathrm{ZY}}\sin\varphi_{z}-\alpha_{\mathrm{ZX}}\cos\varphi_{z}+\alpha_{\mathrm{ZX}}\\ \alpha_{\mathrm{ZY}}\cos\varphi_{z}-\alpha_{\mathrm{ZX}}\sin\varphi_{z}-\alpha_{\mathrm{ZY}} & -\alpha_{\mathrm{ZY}}\sin\varphi_{z}-\alpha_{\mathrm{ZX}}\cos\varphi_{z}+\alpha_{\mathrm{ZX}} & 1 \end{array}\right].$$

Assume the attitude matrices calculated by the navigation solution of the hybrid inertial navigation system before and after rotation are as follows:

(19) $${\tilde{C}}_{n}^{s}{\left(C_{n}^{s}\right)}^{-1}=\left[\begin{array}{ccc} C_{11} & C_{12} & C_{13}\\ C_{21} & C_{22} & C_{23}\\ C_{31} & C_{32} & C_{33} \end{array}\right]$$

Then the non-orthogonality angles *α_ZY_* and *α_ZX_* can be obtained, as shown in Equation (20).

(20) $$\alpha_{\mathrm{ZY}}=\frac{C_{13}+C_{31}}{2\left(\cos\varphi_{z}-1\right)}\;\;\alpha_{\mathrm{ZX}}=\frac{C_{23}+C_{32}}{2\left(1-\cos\varphi_{z}\right)}$$

When the rotation angles of the platform axis and outer gimbal axis are both 0°, i.e., *φ_z_* = *φ_y_* = 0°, and only the inner gimbal axis is rotated to a certain angle, we obtain

(21) $${\tilde{C}}_{n}^{s}{\left(C_{n}^{s}\right)}^{-1}={{R^{\prime }}_{z}}^{-1}{{R^{\prime }}_{x}}^{-1}R_{x}\left(\varphi_{x}\right){R^{\prime }}_{x}{R^{\prime }}_{z}$$

Then the non-orthogonality angles *β_XZ_* and *β_XY_* can be obtained, as shown in Equation (22).

(22) $$\beta_{\mathrm{XZ}}=\frac{C_{12}+C_{21}}{2\left(\cos\varphi_{x}-1\right)}\;\;\beta_{\mathrm{XY}}=\frac{C_{13}+C_{31}}{2\left(1-\cos\varphi_{x}\right)}-\alpha_{\mathrm{ZY}}$$

When the rotation angles of the platform axis and inner gimbal axis are both 0°, i.e., *φ_z_* = *φ_x_* = 0, and only the outer gimbal axis is rotated to a certain angle, we obtain

(23) $${\tilde{C}}_{n}^{s}{\left(C_{n}^{s}\right)}^{-1}={{R^{\prime }}_{z}}^{-1}{{R^{\prime }}_{x}}^{-1}{{R^{\prime }}_{y}}^{-1}R_{y}\left(\varphi_{y}\right){R^{\prime }}_{y}{R^{\prime }}_{x}{R^{\prime }}_{z}$$

The non-orthogonality angles *θ_YZ_* and *θ_YX_* can then be solved, as shown in Equation (24).

(24) $$\theta_{\mathrm{YZ}}=\frac{C_{12}+C_{21}}{2\left(1-\cos\varphi_{y}\right)}-\beta_{\mathrm{XZ}}\;\;\theta_{\mathrm{YX}}=\frac{C_{23}+C_{32}}{2\left(\cos\varphi_{y}-1\right)}-\alpha_{\mathrm{ZX}}$$

In summary, the three-position self-calibration method is conceptually clear, computationally inexpensive, and independent of the initial mounting attitude. However, it requires the system to follow a fixed “static–rotate–static” procedure and is therefore incompatible with arbitrary rotation schedules. It is also sensitive to sensor measurement noise (accelerometer noise and gyroscope angular random walk) because it discards the data collected during rotation and thus exhibits limited robustness.

### 2.5. Recursive Least Squares (RLSs) Calibration Method

To overcome the poor noise immunity of the three-position method and to make full use of the data collected during rotation, this paper proposes a frame non-orthogonality angle calibration method based on recursive least squares (RLSs). The method recasts calibration as a state-estimation problem. Accounting for the non-orthogonality angles of the HINS, the body angular velocity can be written as

(25) $$\begin{array}{rcl} \omega_{\mathrm{nb}}^{b} & = & \omega_{\mathrm{ib}}^{b}-\omega_{\mathrm{in}}^{b}=C_{s}^{b}\omega_{\mathrm{is}}^{s}-\omega_{\mathrm{bs}}^{b}-C_{n}^{b}\omega_{\mathrm{ie}}^{n}\\ & = & R_{y}\left(\varphi_{y}\right)\left[{R^{\prime }}_{y}R_{x}\left(\varphi_{x}\right)\left[{R^{\prime }}_{x}R_{z}\left(\varphi_{z}\right)\left[{R^{\prime }}_{z}\left[\begin{array}{c} \omega_{x}^{s}\\ \omega_{y}^{s}\\ \omega_{z}^{s} \end{array}\right]-\left[\begin{array}{c} 0\\ 0\\ \Omega_{z} \end{array}\right]\right]-\left[\begin{array}{c} \Omega_{x}\\ 0\\ 0 \end{array}\right]\right]-\left[\begin{array}{c} 0\\ \Omega_{y}\\ 0 \end{array}\right]\right]\\ & - & \left[\begin{array}{ccc} {}* & -\mathrm{cos\gamma sin\psi}+\mathrm{sin\gamma sin\theta cos\psi} & -\mathrm{sin\gamma cos\theta}\\ {}* & \mathrm{cos\theta cos\psi} & \mathrm{sin\theta}\\ {}* & -\mathrm{sin\gamma sin\psi}-\mathrm{cos\gamma sin\theta cos\psi} & \mathrm{cos\gamma cos\theta} \end{array}\right]\left[\begin{array}{c} 0\\ \omega_{\mathrm{ie}}\mathrm{cosL}\\ \omega_{\mathrm{ie}}\mathrm{sinL} \end{array}\right] \end{array}$$

Here, the first term is the measured gyroscope angular velocity, and the second is the frame-axis rotation rate obtained from the angle encoders. Because the carrier is stationary, the true body angular velocity should be zero. The method in Ref. [11] requires the INS to be leveled approximately with the local geographic frame so that the attitude-error differential equations can be simplified. To remove the need for an external high-precision horizontal reference and achieve arbitrary-position self-calibration, we introduce the Euler-angle differential kinematic equations. Let the rotation order from the navigation frame to the body frame be Z–X–Y, with the pitch, roll, and heading angles defined as described above, and let counterclockwise rotation be positive. The relation between the body angular velocity and the Euler-angle rates is then given by

(26) $$\left[\begin{array}{c} \omega_{\mathrm{nbx}}^{b}\\ \omega_{\mathrm{nby}}^{b}\\ \omega_{\mathrm{nbz}}^{b} \end{array}\right]=R_{y}\left(\gamma \right)R_{x}\left(\theta \right)\left[\begin{array}{c} 0\\ 0\\ \dot{\psi } \end{array}\right]+R_{y}\left(\gamma \right)\left[\begin{array}{c} \dot{\theta }\\ 0\\ 0 \end{array}\right]+\left[\begin{array}{c} 0\\ \dot{\gamma }\\ 0 \end{array}\right]=\left[\begin{array}{ccc} \mathrm{cos\gamma} & 0 & \mathrm{sin\gamma cos\theta}\\ 0 & 1 & -\mathrm{sin\theta}\\ -\mathrm{sin\gamma} & 0 & \mathrm{cos\gamma cos\theta} \end{array}\right]\left[\begin{array}{c} \dot{\theta }\\ \dot{\gamma }\\ \dot{\psi } \end{array}\right]$$

(27) $$\begin{array}{rcl} \left[\begin{array}{c} \dot{\theta }\\ \dot{\gamma }\\ \dot{\psi } \end{array}\right] & = & {\left[\begin{array}{ccc} \mathrm{cos\gamma} & 0 & \mathrm{sin\gamma cos\theta}\\ 0 & 1 & -\mathrm{sin\theta}\\ -\mathrm{sin\gamma} & 0 & \mathrm{cos\gamma cos\theta} \end{array}\right]}^{-1}\left[\begin{array}{c} \omega_{\mathrm{nbx}}^{b}\\ \omega_{\mathrm{nby}}^{b}\\ \omega_{\mathrm{nbz}}^{b} \end{array}\right]\\ & = & \frac{1}{\mathrm{cos\theta}}\left[\begin{array}{ccc} \mathrm{cos\gamma cos\theta} & 0 & -\mathrm{sin\gamma cos\theta}\\ \mathrm{sin\gamma sin\theta} & \mathrm{cos\theta} & \mathrm{cos\gamma sin\theta}\\ \mathrm{sin\gamma} & 0 & \mathrm{cos\gamma} \end{array}\right]\left[\begin{array}{c} \omega_{\mathrm{nbx}}^{b}\\ \omega_{\mathrm{nby}}^{b}\\ \omega_{\mathrm{nbz}}^{b} \end{array}\right] \end{array}$$

Because the HINS base remains stationary throughout calibration, the external attitude does not vary with time, and the initial attitude angles are fixed constants. The nonlinear cross-coupling matrix can therefore be treated as a constant-coefficient matrix during integration, and the integrated attitude angles in the navigation frame are given below. However, because the Euler-angle equations contain trigonometric functions, the formulation becomes singular when the pitch angle reaches 90°. The method is therefore not applicable to an initial attitude near this singular position.

(28) $$\left[\begin{array}{c} \Delta \theta \\ \Delta \gamma \\ \Delta \psi \end{array}\right]=M_{0}\int \left[\begin{array}{c} \delta \omega_{\mathrm{nbx}}^{b}\\ \delta \omega_{\mathrm{nby}}^{b}\\ \delta \omega_{\mathrm{nbz}}^{b} \end{array}\right]\;\mathrm{dt}+\left[\begin{array}{c} \theta_{0}\\ \gamma_{0}\\ \psi_{0} \end{array}\right]$$

The calibration rotation scheme of the RLS calibration method is consistent with that of the three-position self-calibration method, i.e., the hybrid inertial navigation system is rotated about the platform axis, inner gimbal axis, and outer gimbal axis, respectively. When rotating only about the platform z-axis, according to the gyroscope mathematical model in Section 2.2, the expression can be approximated as shown in Equation (29).

(29) $$\left\{\begin{array}{l} {\tilde{\omega }}_{x}^{s}=\theta_{\mathrm{gx}}^{\mathrm{el}}\omega_{s}+\varepsilon_{\mathrm{gx}}\\ {\tilde{\omega }}_{y}^{s}=\theta_{\mathrm{gy}}^{\mathrm{el}}\omega_{s}+\varepsilon_{\mathrm{gy}}\\ {\tilde{\omega }}_{z}^{s}=\left(K_{\mathrm{gz}}+1\right)\omega_{s}+\varepsilon_{\mathrm{gz}} \end{array}\right.$$

Since the navigation error caused by the gyroscope constant drift is negligible compared with that caused by the frame non-orthogonality angles, we neglect the gyroscope constant drift and higher-order small quantities, as well as the Earth’s rotation angular velocity. This assumption is justified for medium-to-high grade FOGs (angular random walk on the order of 0.0004°/$\sqrt{h}$ and bias instability well below 0.01°/h), whose constant drift is pre-calibrated to the arc-second level before frame non-orthogonality calibration; its contribution to the attitude observations over the 210 s rotation sequence is below 1% of the non-orthogonality-induced errors. Substituting Equation (29) into Equation (28), the body angular velocity ${\tilde{\omega }}_{\mathrm{nb}}^{b}$ can be calculated. 

To quantify the coupling with residual installation errors, we conducted additional simulations with residual gyro installation errors of up to 5″. The resulting bias in the estimated non-orthogonality angles is below 0.3″, which is negligible compared with the estimated angles themselves. Therefore, residual installation errors after system-level calibration do not significantly bias the non-orthogonality estimates.

(30) $$\begin{array}{l} {\tilde{\omega }}_{\mathrm{nbx}}^{b}=\cos\varphi_{z}\left(\theta_{\mathrm{gx}}^{\mathrm{el}}\omega_{s}+\alpha_{\mathrm{ZY}}\omega_{s}\right)-\sin\varphi_{z}\left(\theta_{\mathrm{gy}}^{\mathrm{el}}\omega_{s}-\alpha_{\mathrm{ZX}}\omega_{s}\right)\\ {\tilde{\omega }}_{\mathrm{nby}}^{b}=\sin\varphi_{z}\left(\theta_{\mathrm{gx}}^{\mathrm{el}}\omega_{s}+\alpha_{\mathrm{ZY}}\omega_{s}\right)+\cos\varphi_{z}\left(\theta_{\mathrm{gy}}^{\mathrm{el}}\omega_{s}-\alpha_{\mathrm{ZX}}\omega_{s}\right)\\ {\tilde{\omega }}_{\mathrm{nbz}}^{b}=\left(K_{\mathrm{gz}}+1\right)\omega_{s}+\varepsilon_{\mathrm{gz}}-\Omega_{z} \end{array}$$

Let *ω_s_* be the ideal frame rotation angular velocity; then, after integration, the pitch and roll angles in the body frame can be obtained.

(31) $$\begin{array}{l} \theta =\sin\varphi_{z}\left(\theta_{\mathrm{gx}}^{\mathrm{el}}+\alpha_{\mathrm{ZY}}\right)-\left(1-\cos\varphi_{z}\right)\left(\theta_{\mathrm{gy}}^{\mathrm{el}}-\alpha_{\mathrm{ZX}}\right)+\theta_{0}\\ \gamma =\left(1-\cos\varphi_{z}\right)\left(\theta_{\mathrm{gx}}^{\mathrm{el}}+\alpha_{\mathrm{ZY}}\right)+\sin\varphi_{z}\left(\theta_{\mathrm{gy}}^{\mathrm{el}}-\alpha_{\mathrm{ZX}}\right)+\gamma_{0} \end{array}$$

Similarly, when rotating only about the inner gimbal axis (x-axis), the roll and heading angles in the body frame obtained after integration are as follows:

(32) $$\begin{array}{l} \gamma =\sin\varphi_{x}\left(\theta_{\mathrm{gy}}^{\mathrm{he}}+\beta_{\mathrm{XZ}}\right)+\left(1-\cos\varphi_{x}\right)\left(\alpha_{\mathrm{ZY}}+\beta_{\mathrm{XY}}+\theta_{\mathrm{gz}}^{\mathrm{el}}\right)+\gamma_{0}\\ \psi =\left(1-\cos\varphi_{x}\right)\left(\theta_{\mathrm{gy}}^{\mathrm{he}}+\beta_{\mathrm{XZ}}\right)-\sin\varphi_{x}\left(\alpha_{\mathrm{ZY}}+\beta_{\mathrm{XY}}+\theta_{\mathrm{gz}}^{\mathrm{el}}\right)+\psi_{0} \end{array}$$

Similarly, when rotating only about the outer gimbal axis (y-axis), the pitch and heading angles in the body frame obtained after integration are shown in Equation (33).

(33) $$\begin{array}{l} \theta =\left(1-\cos\varphi_{y}\right)\left(\theta_{\mathrm{YX}}+\alpha_{\mathrm{ZX}}+\theta_{\mathrm{gz}}^{\mathrm{he}}\right)-\sin\varphi_{y}\left(\theta_{\mathrm{YZ}}+\beta_{\mathrm{XZ}}-\theta_{\mathrm{gx}}^{\mathrm{he}}\right)+\theta_{0}\\ \psi =\sin\varphi_{y}\left(\theta_{\mathrm{YX}}+\alpha_{\mathrm{ZX}}+\theta_{\mathrm{gz}}^{\mathrm{he}}\right)+\left(1-\cos\varphi_{y}\right)\left(\theta_{\mathrm{YZ}}+\beta_{\mathrm{XZ}}-\theta_{\mathrm{gx}}^{\mathrm{he}}\right)+\psi_{0} \end{array}$$

Based on the above analysis, there exists a coupling relationship between the six frame non-orthogonality angles and the gyroscope installation errors. Assuming that the gyroscope installation errors have been compensated, the RLS method is used to optimally estimate the frame non-orthogonality angles. A six-dimensional state vector is defined as follows, corresponding to the six frame non-orthogonality angles, respectively.

Under the proposed three-position rotation sequence, the accumulated observation matrix *H_total_* has full column rank (rank = 6) with a condition number of approximately 1.33, confirming that all six angles are observable and well-conditioned. During the rotation sequence, the rank evolves from 2 after the z-axis rotation to 4 after the x-axis rotation, and it reaches 6 only after the y-axis rotation, confirming that all three axes are necessary to fully excite the six non-orthogonality angles; the subsequent reverse rotations only add redundant observations. Note that during a single rotation segment, the RLS state vector contains composite parameters (linear combinations of the individual non-orthogonality angles) rather than the physical angles directly. However, after the full three-position rotation sequence, the accumulated observation matrix spans the full 6-dimensional space. The six physical non-orthogonality angles are then recovered directly from the RLS estimate *X* by a fixed linear inversion: *α_ZY_* = *X*_1_, *α_ZX_* = *X*_2_, *β_XZ_* = *X*_3_, *β_XY_* = *X*_4_ − *X*_1_, *θ_YX_* = *X*_5_ − *X*_2_, *θ_YZ_* = *X*_6_ − *X*_3_.

(34) $$X={\left[\alpha_{\mathrm{ZY}}\;\alpha_{\mathrm{ZX}}\;\beta_{\mathrm{XZ}}\;\alpha_{\mathrm{ZY}}+\beta_{\mathrm{XY}}\;\theta_{\mathrm{YX}}+\alpha_{\mathrm{ZX}}\;\theta_{\mathrm{YZ}}+\beta_{\mathrm{XZ}}\right]}^{T}$$

Substituting the first-order angular-velocity residuals induced by frame non-orthogonality (Equation (30)) into the Euler-angle kinematic Equation (26), integrating from the zero-angle reference, and subtracting the theoretical attitude under zero non-orthogonality yields the residual attitude vector $z_{k}=\begin{array}{ccc} \left[\Delta \mathrm{pitc}h_{k}\right. & \Delta \mathrm{rol}l_{k} & {\left.\Delta \mathrm{headin}g_{k}\right]}^{T} \end{array}$ at discrete time k. This residual is linearly related to the composite parameter vector *X* defined in Equation (34) through the 3 × 6 observation matrix *H_k_* derived from the first-order expansion of Equations (31)–(33), i.e., *z_k_* = *M*_0_*H_k_X* + *v_k_*, where *M*_0_ is the initial coordinate transformation matrix that projects the integrated attitude errors into the navigation frame, and *v_k_* is the measurement noise. The six physical non-orthogonality angles are recovered from *X* via the fixed linear inversion given above. Because this observation equation is derived directly from the Euler-angle kinematic equations without assuming a leveled attitude condition, no external horizontal reference is required; this is in contrast to Ref. [11], which requires the INS to be approximately leveled.

Based on the above derivation, the observation equation can be expressed as

(35) $$\begin{array}{cc} z_{k}=A_{k}X+v_{k} & A_{k}=M_{0}H_{k} \end{array}$$

(36) $$H_{k}=\left[\begin{array}{cccccc} \sin\varphi_{z} & 1-\cos\varphi_{z} & 0 & 0 & 1-\cos\varphi_{y} & -\sin\varphi_{y}\\ 1-\cos\varphi_{z} & -\sin\varphi_{z} & \sin\varphi_{x} & 1-\cos\varphi_{x} & 0 & 0\\ 0 & 0 & 1-\cos\varphi_{x} & -\sin\varphi_{x} & \sin\varphi_{y} & 1-\cos\varphi_{y} \end{array}\right]$$

Here, the measurement vector is the pitch, roll, and heading angles projected into the navigation frame after the navigation solution at discrete time index k (k = 1, 2, 3, …), with a sampling interval of 10 ms; *z_k_* is the residual attitude vector relative to the ideal orthogonal-frame solution, and *A_k_* is the 3 × 6 observation matrix at time k. In summary, the proposed RLS method provides strong noise immunity and stable estimates. By recursively fusing all observations collected during the entire calibration, it suppresses the effect of random noise on individual samples, substantially reducing the fluctuation of the final calibration results and yielding more reliable estimates of the non-orthogonality angles.

The RLS estimator recursively updates the state *X_k_* at each discrete time k as follows: ${\hat{X}}_{k}={\hat{X}}_{k-1}+K_{k}(z_{k}-A_{k}{\hat{X}}_{k-1})$, $K_{k}=P_{k-1}A_{k}^{T}{\left(\mathrm{\lambda I}+A_{k}P_{k-1}A_{k}^{T}\right)}^{-1}$, *P_k_* = (*I* − *K_k_A_k_*)*P_k_*_−1_/*λ*, where *λ* is the forgetting factor (set to 0.998 in this work), *P*_0_ = *αI* with *α* = 10^4^, and the stopping criterion is that the norm of the state update falls below 10^−8^ for 100 consecutive steps.

## 3. Results and Discussion

### 3.1. Non-Orthogonality Calibration Simulation Experiment

**Figure 3:**
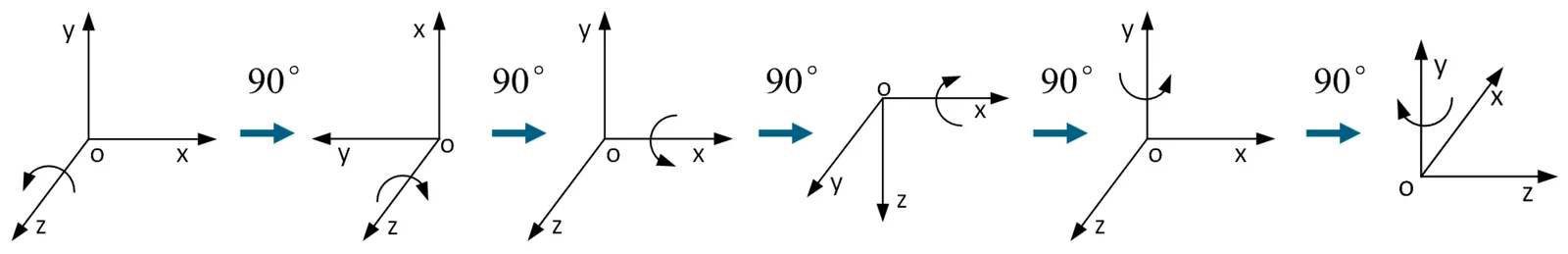
Rotation scheme for frame non-orthogonality calibration.

To verify the effectiveness, accuracy, and convergence of the proposed calibration methods, simulations are conducted. Other inertial sensor errors are assumed to have been compensated by system-level calibration, leaving only residual errors drawn from a zero-mean Gaussian distribution; the parameters are listed in Table 2. The true values of the six non-orthogonality angles are set to 20″, 20″, 30″, 30″, 40″, and 40″. The rotation schedule follows the three-position scheme: 10 s static, +90° rotation about each of the platform, inner-gimbal, and outer-gimbal axes, 20 s static, −90° rotation, and another 10 s static, as shown in Figure 3. The rotation rate is 6°/s and the total calibration time is 210 s.

**Figure 4:**
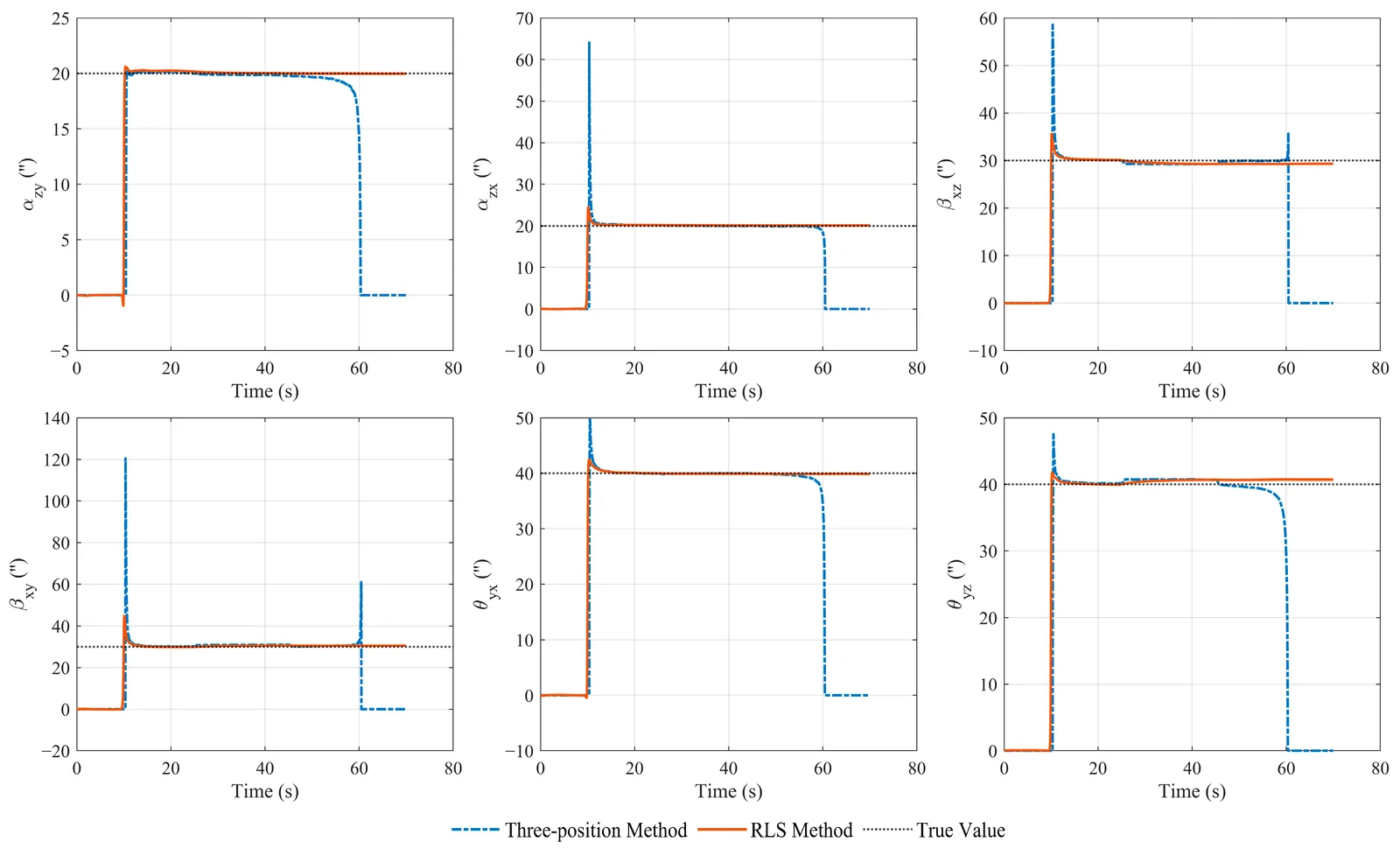
Comparison diagram of frame non-orthogonality error calibration.

Both the three-position and RLS methods are applied to estimate the six non-orthogonality angles; a single-trial result is shown in Figure 4. The black dashed lines are the true values, the blue dash-dot lines are the three-position estimates, and the red solid lines are the RLS estimates. Both methods converge to near the true values, but the RLS estimates converge more smoothly and faster, with smaller fluctuations. The three-position method shows large peaks when the rotation angle passes through zero, because the non-orthogonality errors are observable only during rotation.

**Figure 5:**
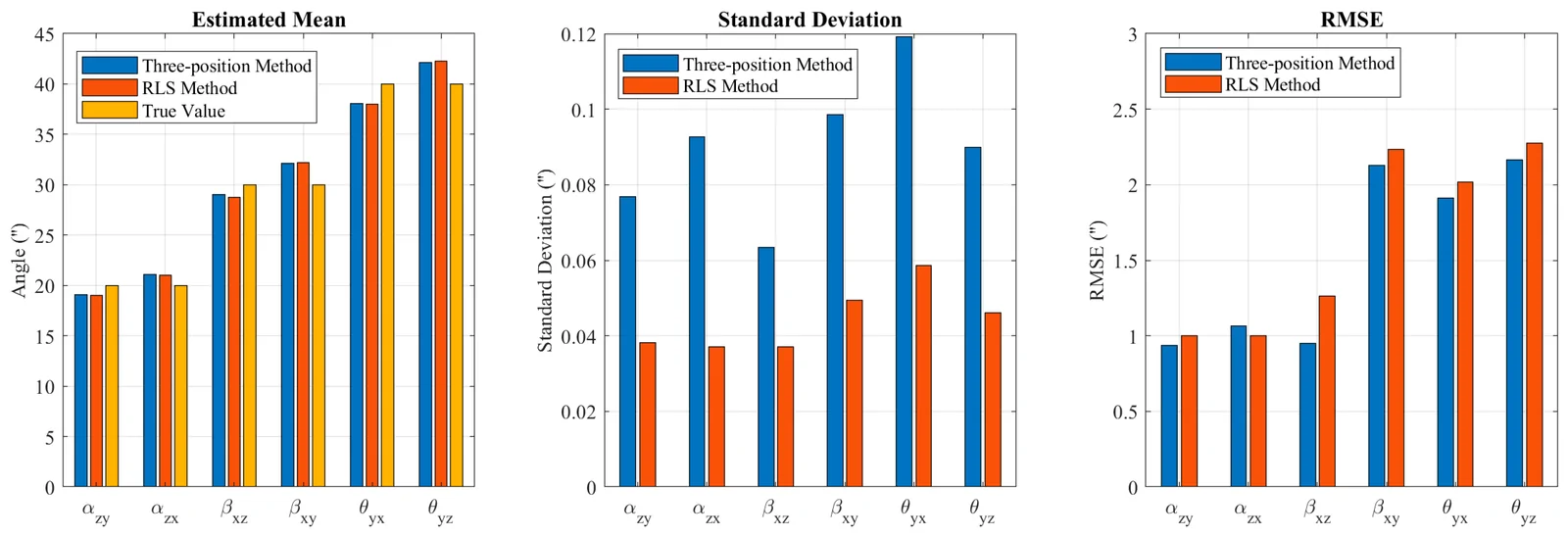
Statistical comparison diagram of non-orthogonality error calibration results.

To assess the effect of random noise, 100 independent Monte Carlo runs are performed. The mean, standard deviation, and root mean square error (RMSE) of the six estimated angles are summarized in Figure 5 and Table 3. In terms of accuracy, the three-position method is slightly better, with an average RMSE of 1.5261″ versus 1.6310″ for RLS; for one particular angle, the three-position RMSE is 0.9497″ versus 1.2608″. This is because the RLS formulation couples gyroscope installation errors with non-orthogonality errors, which means residual gyro installation errors can be absorbed into the non-orthogonality estimates. As shown in Section 2.5, this coupling introduces a small bias rather than providing an unambiguous advantage. In terms of stability and repeatability, RLS is clearly superior: its average standard deviation is only 0.044″, less than half of the 0.0901″ from the three-position method, and its standard deviations are smaller for all six angles.

Overall, the three-position method is suitable for platforms with limited computing power and for initial attitudes away from the ±90° pitch singularity, but it has poor repeatability and stability. The RLS method is better suited to applications that demand high stability and repeatability, particularly in production environments where repeated calibrations must yield consistent results.

It is important to distinguish estimation accuracy (bias) from repeatability (standard deviation). The three-position method achieves comparable average RMSE but with larger run-to-run variation, whereas the RLS method trades a small increase in average RMSE for substantially improved repeatability. The three repeated physical calibrations in Section 3.3 provide limited statistical evidence; the 100-run Monte Carlo results in this section are the primary basis for the repeatability comparison.

In practice, the carrier cannot always be assumed to rest on a perfectly horizontal reference. The RLS method proposed here uses the Euler-angle differential equations and therefore removes the need for an external high-precision horizontal reference, allowing calibration to start from any initial static attitude. As shown in Section 2.5, however, the Euler-angle coupling matrix becomes singular when the initial pitch approaches ±90°. The initial heading does not enter the differential equations and has no effect on calibration. To evaluate the robustness of the algorithm over the full non-singular attitude range, the following attitude-domain tests are designed.

To further validate the proposed RLS method against existing approaches, we implemented the method of Gao et al. [11] under comparable simulation conditions (same true angles, sensor noise, sampling rate, and 100 Monte Carlo runs). The results are summarized in Table 3. The three methods achieve comparable average RMSE (1.53″ for three-position, 1.63″ for our RLS, and 1.50″ for Gao et al.), which is expected because all three methods are based on the same error model and operate under identical noise conditions. However, the key difference lies in the standard deviation (repeatability): our RLS method achieves an average standard deviation of 0.044″, which is approximately 51% lower than the three-position method (0.090″) and approximately 70% lower than Gao et al.’s two-step method (0.145″). In addition, Gao et al.’s velocity-based two-step method exhibits a systematic bias of approximately 1″ for the inner-middle and inner-outer angles due to coupling with east velocity error terms, whereas our attitude-based RLS formulation is immune to this coupling. These results confirm that the proposed RLS method provides significantly better repeatability and avoids the specific velocity-error coupling present in Gao et al.’s method under comparable conditions.

**Figure 6:**
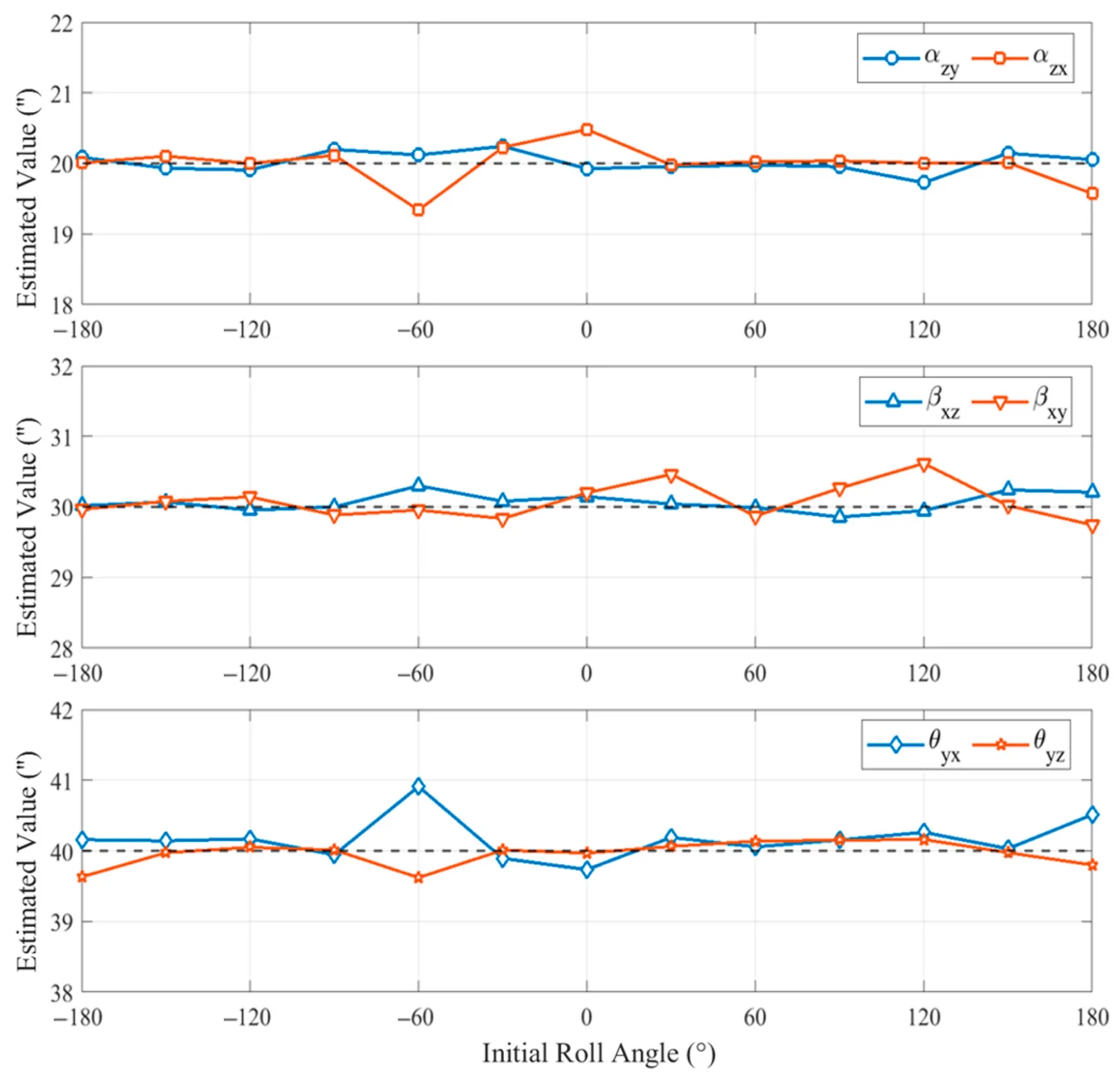
Calibration results under different initial roll angles.

**Figure 7:**
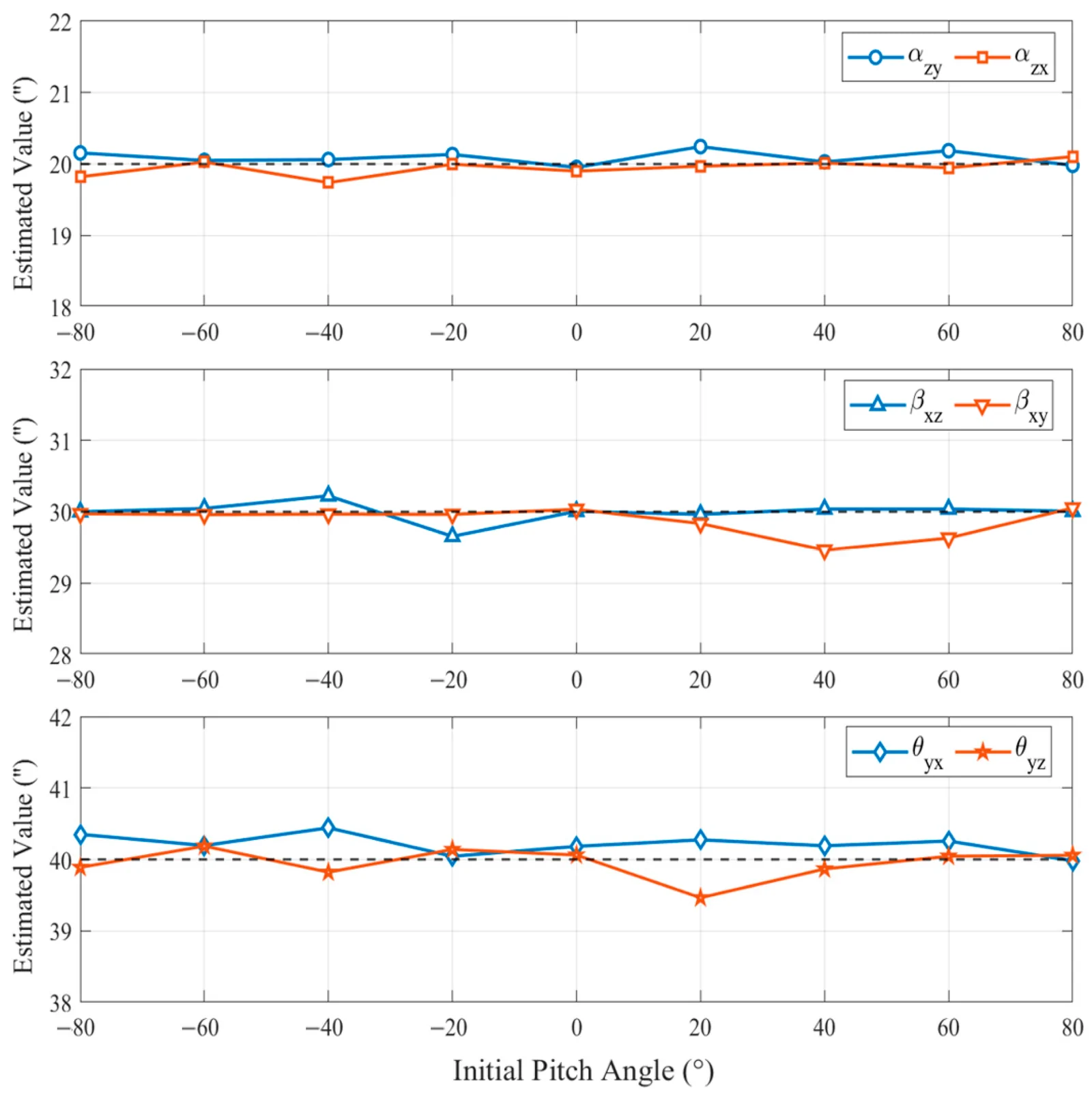
Calibration results under different initial pitch angles.

First, the initial roll is varied in 30° steps while the initial pitch and heading are held at 0°. The residual sensor errors in Table 2 are injected, and the gyro installation errors are set to zero so that non-orthogonality errors are identified independently. Figure 6 shows that all six estimated angles converge stably to the true values within 2″, with no divergence, regardless of the initial roll. Next, the initial pitch is varied in 20° steps within the non-singular range, with roll and heading at 0°. Figure 7 shows that all six estimates again converge within 1″. Thus, within the non-singular region, the initial pitch attitude does not affect the observability of the error parameters.

### 3.2. Transfer Alignment Simulation Experiment

Transfer alignment provides the initial alignment of a slave INS on a moving carrier by using high-precision information from a master INS or another auxiliary source [20]. It is used for the initialization and correction of INSs on moving vehicles, requiring the master INS to supply position, velocity, or attitude reference. Transfer alignment typically proceeds in two stages: coarse alignment, in which the slave INS directly uses the master-INS reference, and fine alignment, in which the outputs of the master and slave INSs are compared and the slave error parameters are estimated by filtering [21].

Transfer alignment is classified by the type of observation used—attitude, velocity, or position matching—among which the combined “velocity + attitude” scheme is the most common [22]. For a HINS slave with a three-axis physical platform, the attitude angles from the navigation solution must first be transformed from the platform frame to the carrier frame before attitude matching with the master INS. Because the three gimbal axes cannot be installed perfectly orthogonally, non-orthogonality errors bias this transformation and therefore degrade attitude-matching accuracy. In high-precision navigation on maneuvering platforms, arcminute-level attitude errors can significantly degrade navigation accuracy [23]. Compensating for frame non-orthogonality angles is therefore expected to improve transfer alignment accuracy.

To quantify the improvement in transfer alignment accuracy after non-orthogonality compensation, a simulation is set up. Common maneuvers for marine vehicles include speed-varying straight runs and C- and S-shaped turns; an S-shaped trajectory is therefore chosen for the master INS [24], consisting of a straight segment, a left turn, a right turn, and a final straight recovery. The vehicle speed is held at 10 knots for 50 s with a 10 ms sampling interval. To reproduce the acceleration and deceleration during turns, the angular rate in the maneuvering segments follows a sinusoidal law, as given in Equation (37).

(37) $$\omega_{z}(t)=\left\{\begin{array}{ll} 0, & 0\leq t<10\\ 5^{{}^{\circ}}\times \left[1+\sin\left(\frac{2\pi (t-10)}{10}-\frac{\pi }{2}\right)\right], & 10\leq t<20\\ -5^{{}^{\circ}}\times \left[1+\sin\left(\frac{2\pi (t-20)}{10}-\frac{\pi }{2}\right)\right], & 20\leq t<30\\ 0, & 30\leq t\leq 50 \end{array}\right.$$

To ensure reproducibility, the detailed simulation parameters are specified as follows. The carrier starts at 34° N, 108° E with an initial speed of 5.14 m/s. The maneuver profile lasts 50 s: from 0 s to 10 s the vessel travels straight and level; from 10 s to 20 s the vessel executes a left turn with angular velocity varying sinusoidally from 0°/s to 10°/s about the up axis; from 20 s to 30 s the vessel executes a symmetric right turn; from 30 s to 50 s the vessel travels straight and level.

The transfer-alignment filter is a 15-state federated Kalman filter with a state vector: three misalignment angles, three gyro biases, three velocity errors, three accelerometer biases, and three installation misalignment angles. The measurement vector consists of three attitude errors (derived from the master and slave quaternion difference) and three velocity differences. The initial standard deviations are: 10° for misalignment angles, 0.001°/h for gyro biases, 10 m/s for velocity errors, 10 µg for accelerometer biases, and 10° for installation angles. The measurement noise standard deviations are 1′ (60″) for attitude and 0.1 m/s for velocity. The filter runs for the full 50 s; convergence is declared when the attitude error estimate stays within 6″ of the true value.

**Figure 8:**
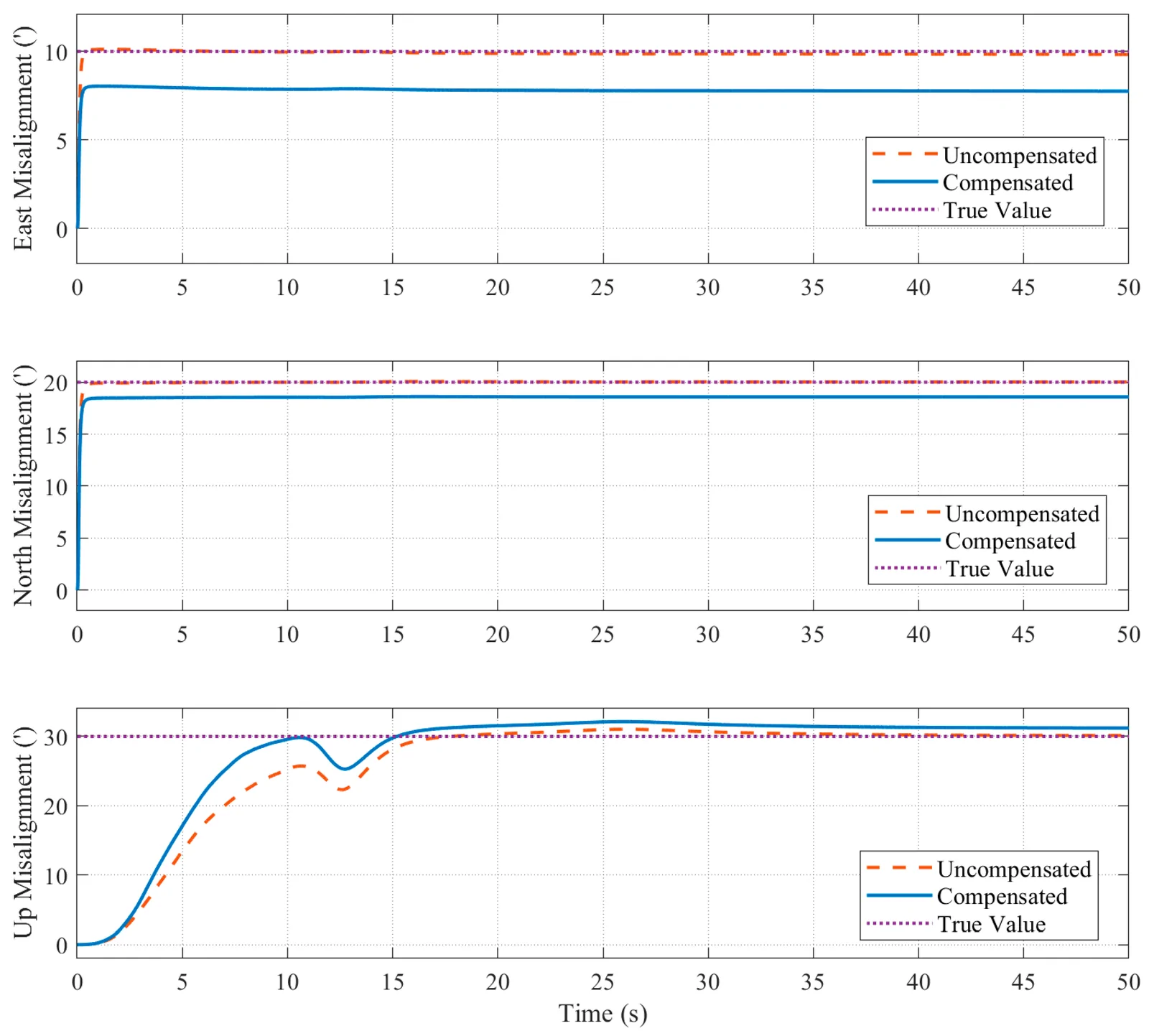
Comparison diagram of misalignment angle convergence curves in transfer alignment.

To construct a simulation environment close to the real system, the frame non-orthogonality angles introduced in this simulation are derived from calibration results of physical experiments. The true values of the east, north, and vertical misalignment angles for transfer alignment are set to 10′, 20′, and 30′, respectively. The physical RLS calibration estimates are applied directly as the compensation parameters, so this simulation represents the ideal case in which the residual calibration error is small relative to the non-orthogonality angles themselves. The frame non-orthogonality errors are calibrated using the RLS calibration method. The convergence curves of the transfer alignment misalignment angle estimates before and after compensation are shown in Figure 8 below, and the specific performance indicators are presented in Table 4.

The simulation shows that transfer alignment accuracy is clearly improved after non-orthogonality compensation: the compensated curves converge faster and with smaller fluctuations. Each of the three misalignment angles improves by at least 0.92′, with a maximum improvement of 2.04′. The two horizontal misalignment angles improve by more than 96%, and the vertical one by more than 80%. Calibrating and compensating for frame non-orthogonality angles therefore removes the systematic errors they introduce and is key to improving the transfer alignment accuracy of a HINS slave.

### 3.3. Physical Experiments

**Figure 9:**
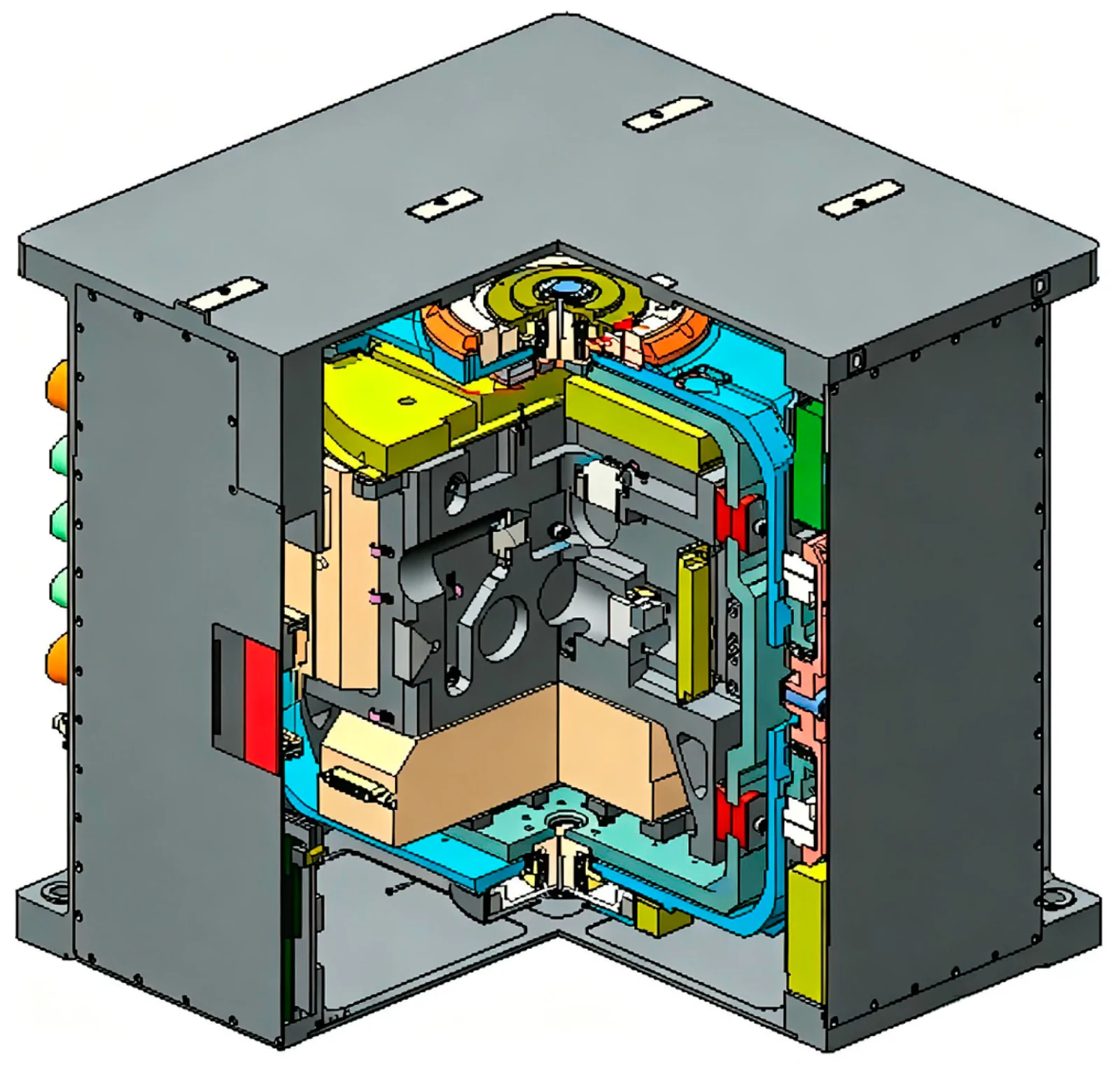
Hybrid inertial navigation system experimental platform.

Physical experiments are conducted on a HINS prototype equipped with a triad of fiber optic gyroscopes (FOGs) and quartz flexible accelerometers. The key sensor noise characteristics are listed in Table 2: the accelerometer random noise is approximately 5 µg and the gyroscope random noise is on the order of 0.0004°/$\sqrt{h}$. The three gimbal frames are driven by brushless motors and read out by angle encoders; the sensor data are sampled at 100 Hz (10 ms interval). A photograph of the experimental platform is shown in Figure 9. Residual inertial sensor errors are pre-calibrated by a system-level Kalman filter.

**Figure 10:**
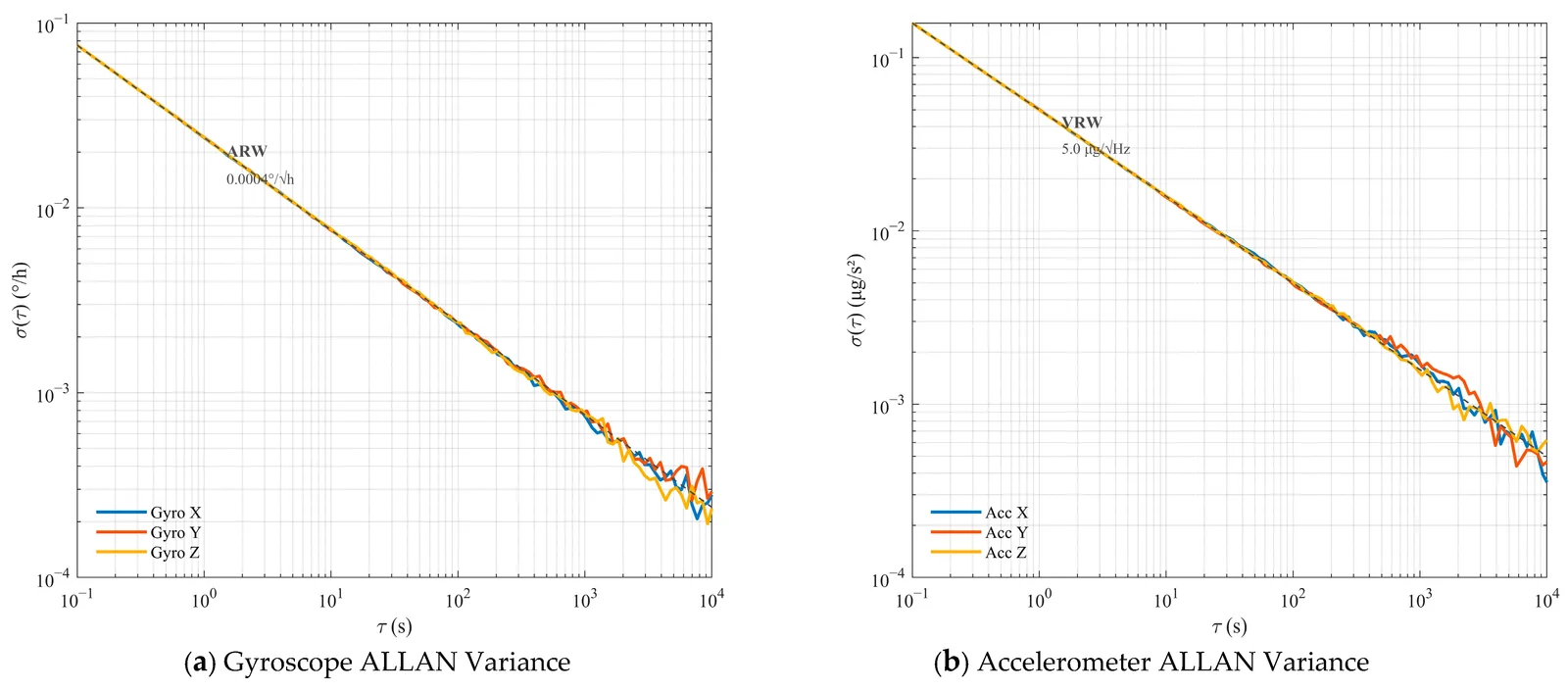
ALLAN deviation curves of the inertial sensors: (**a**) gyroscope and (**b**) accelerometer.

To confirm the sensor noise characteristics, ALLAN variance analysis was performed on the static data collected from the HINS prototype. As shown in Figure 10, the gyroscope angle random walk (ARW) is estimated to be 0.0004°/$\sqrt{h}$, and the accelerometer velocity random walk (VRW) is estimated to be 5.0 µg/$\sqrt{\mathrm{Hz}}$, both consistent with the parameters listed in Table 2. The fixed bias terms are not resolved in the short-duration ALLAN curves due to the dominance of random noise and are taken directly from the calibrated values.

The data window for each static segment is 20 s, with encoder synchronization accuracy of <1 ms. Valid static measurements are selected by checking that the gyro output rate variation is below 0.001°/s. The RLS initialization is *P*_0_ = 10^4^*I* and *θ*_0_ = 0, with the same forgetting factor *λ* = 0.998 as in simulation.

**Figure 11:**
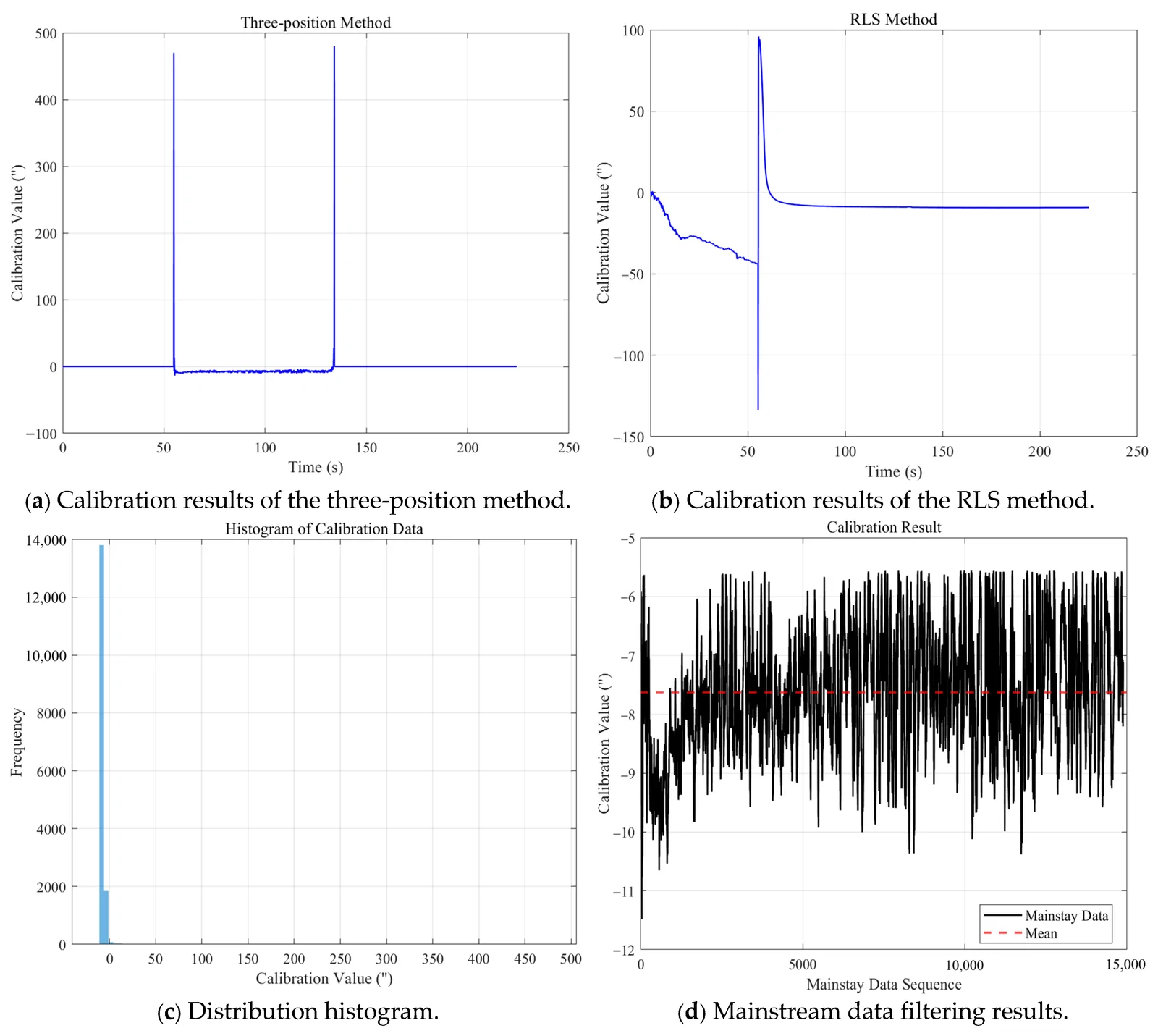
Calibration results of frame non-orthogonality errors (**a**) Calibration results of the three-position method. (**b**) Calibration results of the RLS method. (**c**) Distribution histogram. (**d**) Mainstream data filtering results.

Both the three-position and RLS methods are applied to calibrate the frame non-orthogonality angles. Taking one angle as an example, Figure 11a,b show the results of the two methods. The RLS estimates form a smooth convergence curve as the state is updated recursively, and the final estimate is used directly. The three-position method instead produces an independent estimate at each sampling instant. Under real experimental conditions, environmental noise causes the estimates to fluctuate considerably more than in simulation.

To process the three-position results, a main-interval data filtering strategy is proposed. First, zero-valued samples are removed and a histogram of the remaining data is built. The most frequent interval is selected as the main interval, with a tolerance added at both ends to cover the bulk of the data. Samples falling within this interval are then used to compute the mean and the max–min range, which describe the central tendency and dispersion. This strategy suppresses the effect of outliers and improves the reliability of the estimated parameters.

Figure 11c,d show the histogram and the filtered main-interval data, respectively; the mean of the filtered data is taken as the three-position calibration result. As expected, the three-position estimates fluctuate at every sampling instant, and the main-interval strategy only partly suppresses the outliers. Because the thresholds are chosen manually, the method introduces some uncertainty and cannot reach the optimal estimation performance of the RLS approach, which further limits the final accuracy.

To compare repeatability, three independent calibrations are run with each method. The mean and standard deviation of the estimates are listed in Table 5. Both methods yield consistent results, but on average the RLS method has smaller standard deviations and better stability, although for certain individual parameters (e.g., parameters 1 and 5) the three-position method yields comparable or slightly smaller variation.

Specifically, the two calibration methods show relatively large deviations in the *β_XZ_*, *θ_YX_*, and *θ_YZ_* parameters. From the single calibration results, the maximum difference can reach about 5″, while the differences between the calibration means are all controlled within the range of 0.5″ to 5″. In addition, the standard deviation of the three-position self-calibration method for the *θ_YZ_* angle calibration reaches 5.12″, whereas the RLS calibration method reduces it to 1.87″, significantly improving the estimation stability of this parameter.

On average, the standard deviation across all six angles is 2.68″ for the three-position method and 1.79″ for RLS—an overall reduction of about 33%. The RLS method therefore has better repeatability and is better suited to the tested stationary-carrier conditions.

**Figure 12:**
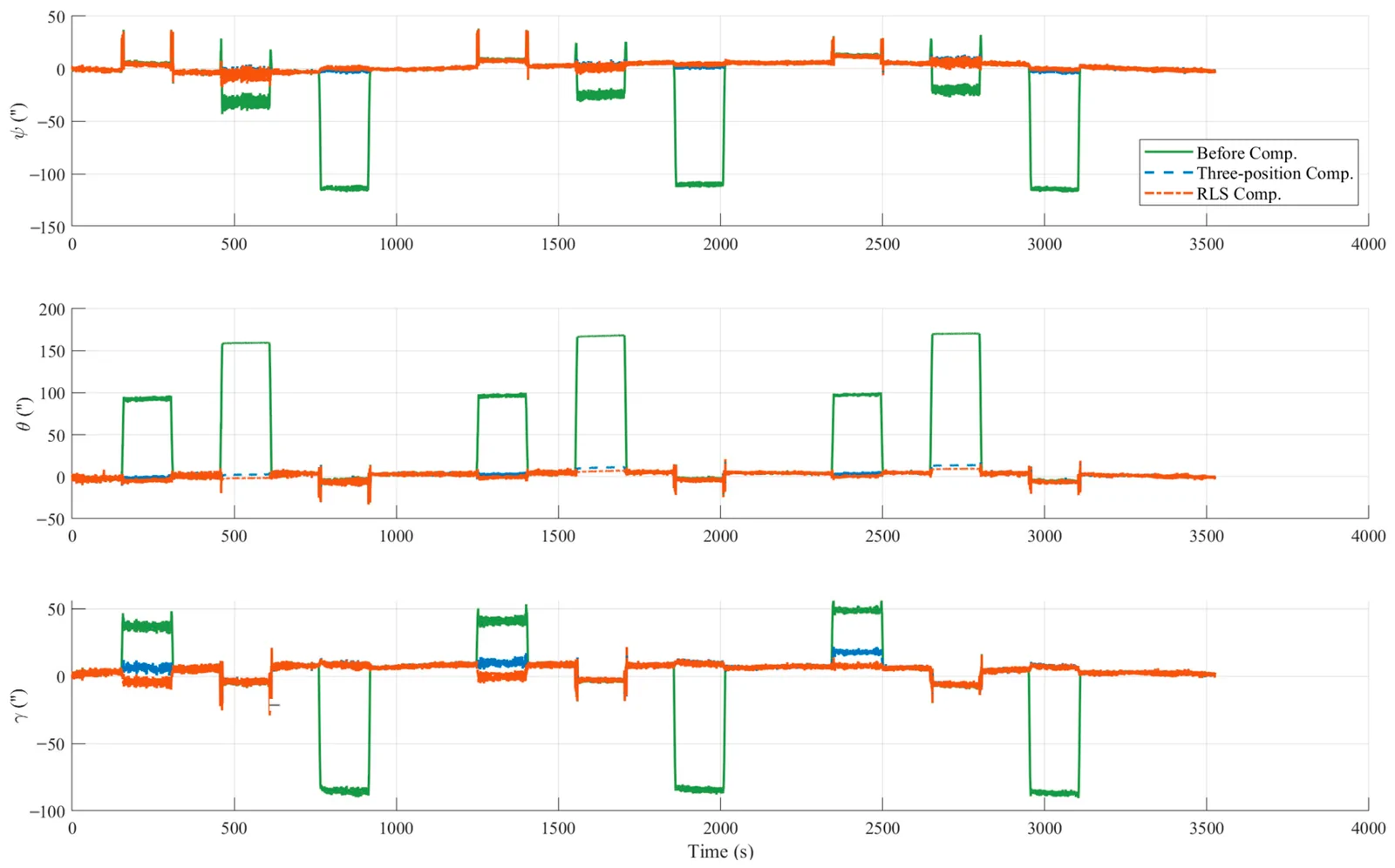
Comparison diagram of attitude errors before and after compensation.

In the physical experiments, the true values of the non-orthogonality angles are not known. Because the carrier remains stationary throughout, the true attitude is constant but is not independently known; the attitude obtained from the navigation solution and projected into the carrier frame can therefore be treated as the attitude error. The attitude errors before and after compensation are compared in Figure 12 and Table 6. As shown in Section 2.3, single-axis rotation excites attitude errors only in the non-rotating axes, so the analysis focuses on those axes.

The initial attitude is determined from the attitude output after coarse alignment on a stationary base; no independent external reference (e.g., a turntable) is available. Therefore, the experimental results are interpreted as repeatability and relative attitude-error reduction rather than absolute calibration accuracy.

After compensation, the attitude errors during rotation are clearly reduced for all axes, confirming that both methods suppress the errors caused by non-orthogonality. The average attitude error is reduced by 82.7″ with the three-position method and by 85.8″ with RLS, with RLS performing slightly better. In particular, for rotation about the y-axis, RLS reduces the roll error by about 10″ more than the three-position method. After three-position compensation, the peak residual errors are within 20″ for all axes; after RLS compensation, the peaks are further reduced, with the peak roll error dropping from 18.53″ to 10.6″.

## 4. Conclusions

This paper focuses on the high-precision calibration of frame non-orthogonality angles in HINS and their application to transfer alignment. A complete error model containing six non-orthogonality angles is established, and two self-calibration schemes are proposed: the three-position method and the RLS method.

Monte Carlo simulations show that the three-position method is simple and computationally cheap but sensitive to sensor measurement noise (accelerometer noise and gyroscope angular random walk), whereas the RLS method recursively fuses the observations collected throughout rotation and offers better noise immunity and repeatability, reducing the average standard deviation of the estimated angles to 0.044″. The RLS method also removes the need for an external high-precision horizontal reference and allows calibration from any static initial attitude within the non-singular region, improving the practical adaptability of the scheme.

Transfer alignment simulations confirm that compensating for non-orthogonality angles substantially improves alignment accuracy: the horizontal misalignment angles improve by more than 96% and the vertical angle by more than 80%, effectively removing the attitude transformation errors introduced by imperfectly orthogonal frame mounting. Physical experiments on a HINS prototype further validate the method: the peak residual roll error is 10.6 arcseconds with RLS-based compensation, compared with 18.53 arcseconds with three-position compensation, improving the overall navigation performance of the system.

This work addresses the limitations of existing methods—incomplete modeling, poor noise immunity, and dependence on a specific initial attitude—and provides a practical approach to suppressing non-orthogonality errors and improving the performance of HINS.

## Figures and Tables

**Table 1:** Coordinate frame definitions.

Coordinate System	Description
i	Inertial coordinate system
e	Earth coordinate system
n	Navigation coordinate system
b	Vehicle/Carrier-fixed coordinate frame
s	IMU coordinate system
h1	Platform base coordinate system
h2	Inner gimbal coordinate system
h3	Outer gimbal coordinate system

**Table 2:** Parameter settings of residual inertial sensor errors.

Parameter Name	Value Range
Accelerometer bias	∼*N*(0,(5μg)^2^)
Accelerometer scale factor error	∼*N*(0,(5ppm)^2^)
Accelerometer installation error	∼*N*(0,(2″)^2^)
Accelerometer random noise	5 µg/$\sqrt{h}$
Gyroscope drift	∼*N*(0,(0.002°/h)^2^)
Gyroscope scale factor error	∼*N*(0,(2ppm)^2^)
Gyroscope installation error	∼*N*(0,(1″)^2^)
Gyroscope random noise	0.0004°/$\sqrt{h}$
Sampling rate	100 Hz

**Table 3:** Comprehensive performance index comparison of non-orthogonality error calibration.

Comprehensive Index	Three-Position Calibration Method	RLS Calibration Method	Gao et al. [11]
Average RMSE	1.5261″	1.6310″	1.5017″
Average Standard Deviation	0.0901″	0.044″	0.1447″
Systematic Bias	NO	NO	~1″

**Table 4:** Comparison of transfer alignment accuracy improvement before and after compensation.

Misalignment Angle	Uncompensated Estimate	Compensated Estimate	True Value	Improvement Magnitude	Improvement Percentage
*φ_E_*	7.89′	9.93′	10′	2.04′	96%
*φ_N_*	18.52′	20.03′	20′	1.45′	97%
*φ_U_*	31.14′	30.22′	30′	0.92′	80%

**Table 5:** Comparison of frame non-orthogonality angle calibration results.

Parameter Name	Three-Position Self-Calibration Method		RLS Calibration Method	
	Mean (″)	Standard Deviation (″)	Mean (″)	Standard Deviation (″)
*α_ZY_*	−102.24	0.45	−103.10	1.02
*α_ZX_*	−11.59	3.88	−10.88	1.45
*β_XZ_*	−64.89	3.68	−66.77	3.15
*β_XY_*	9.88	1.94	9.13	0.95
*θ_YX_*	41.17	1.02	38.88	2.31
*θ_YZ_*	−1.62	5.12	2.42	1.87

**Table 6:** Comparison table of statistical performance indicators.

Performance Index	Three-Position Self-Calibration Method			RLS Calibration Method		
	Heading Angle	Pitch Angle	Roll Angle	Heading Angle	Pitch Angle	Roll Angle
Average Attitude Error Reduction	67.1″	120.3″	60.9″	67.7″	123.8″	65.8″
Peak Residual Attitude Error	13.2″	13.6″	18.53″	13.1″	9.5″	10.6″

## Data Availability

The original contributions presented in this study are included in the article. Further inquiries can be directed to the corresponding author.

## Abbreviations

The following abbreviations are used in this manuscript:

INS	Inertial Navigation System
RLS	Recursive Least-Squares
HINS	Hybrid Inertial Navigation System
RMSE	Root Mean Square Error
IMU	Inertial Measurement Unit
SINS	Strapdown Inertial Navigation System
